# Therapeutic Efficacy of 7‑Chloro-4-(phenylselanyl)quinoline against Oncological Pain Induced by Tumor Progression and Vincristine Treatment

**DOI:** 10.1021/acsomega.5c11481

**Published:** 2026-06-12

**Authors:** Ketlyn P. da Motta, Vanessa M. E. da Rocha, Bruna S. Pacheco, Tiago V. Collares, Fabiana K. Seixas, Joanna V. Z. Echenique, Mauro P. Soares, Rhayane Tavares, Diego Alves, Nathalia S. Pedra, Natália P. Bona, Roselia M. Spanevello, Juliano S. Barin, Pricila N. Pinheiro, Vinicius C. Prado, Marcia F. Mesko, Ethel A. Wilhelm

**Affiliations:** † Graduate Program in Biochemistry and Bioprospecting, Preclinical and Translational Research Group on Pain and Chronic Diseases, Center for Chemical, Pharmaceutical, and Food Science, 37902 Federal University of Pelotas (UFPel), 96010-900 Pelotas, RS, Brazil; ‡ Cellular and Molecular Oncology Research Group (GPO), CDTec, Federal University of Pelotas (UFPel), 96010-900 Pelotas, RS, Brazil; § Regional Diagnostic Laboratory Faculty of Veterinary Medicine, Federal University of Pelotas, 96010-900 Pelotas, RS, Brazil; ∥ Graduate Program in Chemistry, Clean Organic Synthesis Laboratory, Federal University of Pelotas (UFPel), 96010-900 Pelotas, RS, Brazil; ⊥ Graduate Program in Biochemistry and Bioprospecting, Laboratory of Neurochemistry, Inflammation and Cancer, 96010-900 Pelotas, RS, Brazil; # Department of Food Science and Technology, Federal University of Santa Maria, 97105-900 Santa Maria, RS, Brazil; ∇ Contaminant Control Laboratory in Biomaterials (LCCBio Federal University of Pelotas (UFPel), 96010-900 Pelotas, RS, Brazil

## Abstract

Cancer pain is a complex condition often worsened by tumor progression and chemotherapy-induced neuropathy. This study evaluated the therapeutic potential of 7-chloro-4-(phenylselanyl)­quinoline (4-PSQ) in mice with Sarcoma 180 (S180)-induced cancer pain and vincristine (VCR)-induced peripheral neuropathy. Male mice were assigned to Control, S180, S180 + VCR, S180 + 4-PSQ, and S180 + VCR + 4-PSQ groups. S180 cells were injected intraplantarly on day 1; VCR was administered days 2–6, and 4-PSQ orally from days 8–17. Nociceptive behavior, tumor progression, and tissue biomarkers were assessed. S180 and VCR caused hyperalgesia, tumor edema, oxidative imbalance, and impaired Na^+^/K^+^/Mg^2+^-ATPase activities. 4-PSQ attenuated hyperalgesia, tumor growth, oxidative stress, and immune dysregulation, restoring ATPase activities. Histology confirmed a tumor mass and inflammatory infiltration. These results indicate that 4-PSQ exerts antinociceptive, antioxidant, and immunomodulatory effects, supporting its potential as an adjuvant therapy for cancer pain and chemotherapy-induced neuropathy.

## Graphical abstract

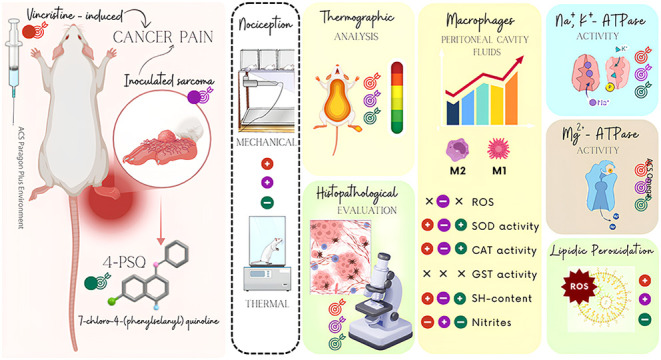

## Introduction

Oncological pain is a complex and debilitating condition that significantly reduces the quality of life for cancer patients.^1–3^ A recent epidemiological study by Snijders et al.^4^ found that, on average, 44.5% of patients experience cancer-related pain, increasing to 54.6% among those in advanced or terminal stages. Although initially thought to result from tumor growth and local invasion, cancer pain actually involves a much more intricate pathophysiology, including ongoing inflammation, nerve injury, and side effects from treatments, especially chemotherapy.^1,2^

Chemotherapeutic agents, although essential for controlling tumors, can paradoxically exacerbate nociceptive processes. Vincristine (VCR), a vinca alkaloid commonly used in cancer treatment due to its strong ability to inhibit cell division, is effective against a wide range of cancers.^5,6^ However, up to 90% of patients treated with VCR develop chemotherapy-induced peripheral neuropathy, a chronic, dose-limiting side effect characterized by axonal degeneration, neuroinflammation, and intense neuropathic pain.^7,8^ These effects not only reduce treatment adherence but also create a significant clinical burden, with few therapeutic options available to address them.

To promote an understanding of the multifactorial mechanisms underlying the modulation and triggering of oncological pain conditions, an experimental investigation of patterns encompassing both the pain associated with tumor development and tissue compression by the tumor, as well as chemotherapy treatment, is necessary. Therefore, in experimental settings, intraplantar inoculation of Sarcoma 180 (S180) cells in rodents has emerged as a well-characterized model to recapitulate cancer-induced pain.^9,10^ This model mimics the main pathological characteristics of tumor progression, including tissue remodeling, inflammatory cell infiltration, and peripheral sensitization^9–11^

Despite the availability of pharmacological options such as opioids, antidepressants (duloxetine), and calcium channel modulators (pregabalin), current treatments are hindered by limited efficacy, tolerance development, and adverse side effects, often leading to treatment-resistant pain.^12,13^ In this therapeutic gap, selenium-based compounds have gained attention for their redox-modulating and neuroprotective properties. Among these, the organoselenium compound 7-chloro-4-(phenylselanyl)­quinoline (4-PSQ) has demonstrated promising preclinical efficacy. Previous studies have demonstrated that 4-PSQ exerts significant effects against neuropathic pain caused by chemotherapy. The pharmacological potential of 4-PSQ is linked to its antioxidant effects, modulation of ATP-dependent enzymes involved in neuronal excitability (including Ca^2+^, Mg^2+^, and Na^+^, K^+^-ATPases), and displays neuroprotective potential.^14–20^ Its antinociceptive properties are likely mediated through anti-inflammatory pathways, including downregulation of pro-inflammatory cytokines (TNF-α, IL-1β), restoration of Nrf2 and NF-κB signaling, and attenuation of mitochondrial dysfunction.^17,18,21,22^

In this study, we aimed to evaluate the antinociceptive potential of 4-PSQ in a murine model of cancer pain induced by S180 tumor inoculation and potentiated by VCR-induced neuropathy. Using a multimodal experimental approach, including behavioral pain assays (mechanical and thermal sensitivities), thermographic imaging, histopathological tumor validation, peritoneal macrophage cytotoxicity analysis, oxidative stress profiling, and enzymatic evaluation of Na^+^, K^+^-ATPase and Mg^2+^-ATPase activities in distinct regions of the central nervous system, we sought to elucidate both peripheral and central mechanisms of 4-PSQ’s pharmacological action. Our findings offer novel insights into the potential of 4-PSQ as a candidate adjuvant therapy for managing cancer- and chemotherapy-induced pain.

## Results and Discussion

This study presents a novel, translationally relevant model of cancer pain combining S180-induced nociception with VCR-induced neurotoxicity, recapitulating mixed pain states in patients. Integrating behavioral, histopathological, thermographic, biochemical, and immunological end points, the work provides a comprehensive mechanistic framework linking tumor progression, inflammation, and chemotherapy-induced neurotoxicity. Thermography offers a noninvasive approach to monitor tumor-associated inflammation, while detailed macrophage profiling highlights the complexity of the immune response. Importantly, 4-PSQ demonstrates analgesic, neuroprotective, antioxidant, and immunomodulatory effects, particularly when combined with VCR, positioning it as a promising candidate for multifaceted cancer pain therapies.

### Assessment of Tumor Progression

Tumor progression in the Sarcoma-induced cancer pain model was quantitatively assessed by monitoring changes in paw width and depth every 48 h (Figure 1). These morphometric parameters, measured with a digital caliper, served as indirect indicators of tumor growth and local edema after intraplantar inoculation of S180 cells and systemic VCR administration. Figure 1A shows tumor progression by measuring the lateral width (latero-lateral dimension) of the animals’ left hind paw. From day 3 to day 17 of the experiment, inoculation of S180 cells caused a steady increase in paw width across all groups receiving tumor induction (S180, S180 + VCR, S180 + 4-PSQ, and S180 + VCR + 4-PSQ). Notably, the S180 group showed significant increases in paw width compared to the control group, with percentage differences of approximately 6% on day 3, 28% on day 5, 64% on day 7, 56% on day 9, 77% on day 11, 104% on day 13, 86% on day 15, and 66% on day 17.

**Figure 1:**
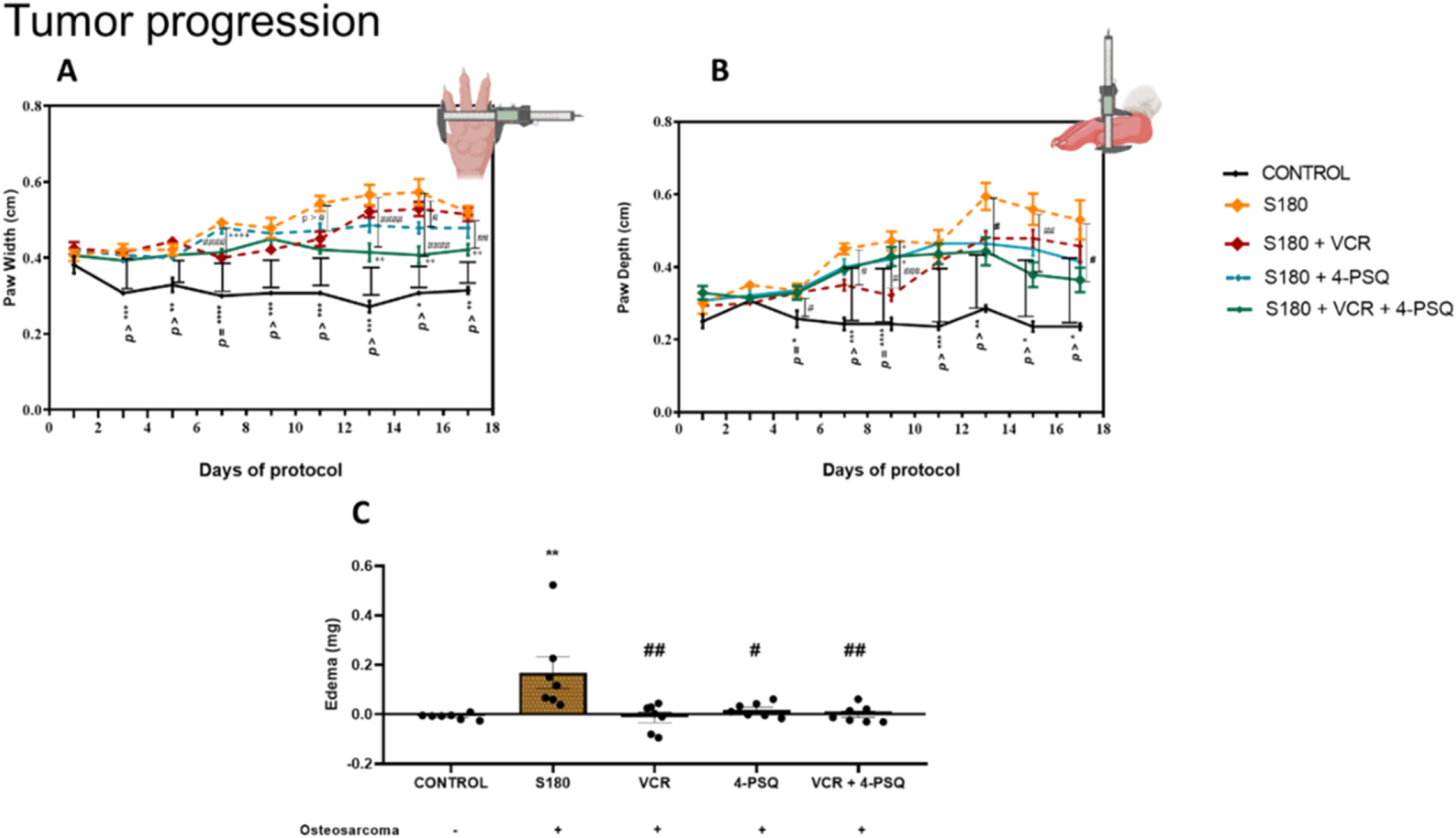
Evaluation of the therapeutic potential of 7-chloro-4-(phenylselanyl)­quinoline (4-PSQ) and vincristine (VCR) against tumor progression of Sarcoma (S180 cells) in a cancer pain model. Left hind paw width (A) and depth (B) were assessed every 2 days throughout the experimental protocol. Tumor-associated paw edema was evaluated as the difference between the weight of the tumor-bearing paw and the contralateral control paw (Δ = control paw weight – tumor paw weight) (C). Each column represents the mean ± standard error of the mean (S.E.M) of 7 animals per group. (*) *P* < 0.05, (**) *P* < 0.01, (***) *P* < 0.001, and (****) *P* < 0.0001 denote significance levels when compared with the control group. (^#^) *P* < 0.05, (^##^) *P* < 0.01, (^###^) *P* < 0.001, and (^####^) *P* < 0.0001 denote significance levels compared with the S180 group. (^+^) *P* < 0.05, (^++^) *P* < 0.01, and (^++++^) *P* < 0.0001 denote significance levels compared with S180 + VCR group. The symbols (<) denote that the *p*-value is equal to or higher than the significance. One-way ANOVA followed by Tukey’s test was used.

VCR administration attenuated tumor-induced paw swelling on days 7, 11, and 13. Treatment with 4-PSQ alone (S180 + 4-PSQ group) significantly reduced paw width compared to the S180 group on days 11 and 15. Moreover, the combined treatment (S180 + VCR + 4-PSQ) resulted in a sustained reduction in paw width compared to the S180 group on days 7, 11, 13, 15, and 17. When comparing the S180 + VCR group to the S180 + VCR + 4-PSQ group, the addition of 4-PSQ enhanced the therapeutic effect, with greater reductions observed on days 13, 15, and 17.

Figure 1B shows tumor growth through depth measurements of the left hind paw during the experiment. From day 3 to day 17, intraplantar inoculation with S180 cells caused a steady, significant increase in paw depth across all tumor-bearing groups (S180, S180 + VCR, S180 + 4-PSQ, and S180 + VCR + 4-PSQ). Importantly, the S180 group showed notable increases in paw depth compared to the control group, with percentage differences reaching about 14% on day 3, 30% on day 5, 85% on day 7, 94% on day 9, 97% on day 11, 108% on day 13, 137% on day 15, and 125% on day 17.

VCR monotherapy attenuated tumor-induced paw swelling at early time points, with significant reductions observed on days 3, 7, and 9. Treatment with 4-PSQ alone (S180 + 4-PSQ) led to a significant reduction in paw depth only on day 13 compared to the S180 group. Importantly, the combination therapy (S180 + VCR + 4-PSQ) produced a sustained reduction in paw depth from day 13 onward, with significant differences observed on days 13, 15, and 17. Moreover, when comparing the S180 + VCR group to the S180 + VCR + 4-PSQ group, the addition of 4-PSQ enhanced the therapeutic effect on paw depth, reaching statistical significance on day 9.

The assessment of tumor progression through quantitative analysis of paw dimensions proved to be a reliable and sensitive indicator of local tumor development in the Sarcoma model. Both the mediolateral (width) and dorsoventral (depth) measurements of the inoculated paw progressively increased over the course of the experimental protocol, correlating with the expansion of neoplastic tissue and associated inflammatory processes.^23–25^ These changes were particularly marked in animals receiving S180 cell inoculation alone, whereas cotreatment with VCR and/or 4-PSQ attenuated paw enlargement, indicating a therapeutic modulation of tumor growth and/or associated edema.

### Assessment of Tumor-Associated Edema

Paw edema was assessed on day 18 of the experimental protocol, immediately following euthanasia, serving as a terminal end point for evaluating tumor-associated inflammatory swelling. As illustrated in Figure 1C, intraplantar inoculation with S180 Sarcoma cells resulted in marked edema formation and tumor development, evidenced by a substantial increase in the weight of the left hind paw (approximately 1850% relative to the contralateral paw). Monotherapy with either VCR or 4-PSQ, as well as the combined treatment (VCR + 4-PSQ), significantly attenuated paw swelling and contributed to a reduction in tumor burden.

The terminal evaluation of paw edema through comparative mass analysis between the inoculated and contralateral paw further substantiated the inflammatory and tumoral burden. The marked increase in paw weight observed in the S180 group confirmed substantial edema formation, likely reflecting both tumor mass and peritumoral inflammation. Importantly, this end point measurement aligned with the dimensional analyses and was significantly mitigated by all treatment regimens, particularly when VCR and 4-PSQ were administered concurrently, suggesting a synergistic effect in controlling tumor-associated inflammatory swelling. Indeed, our findings are consistent with previous studies that employed S180 cell inoculation to induce solid tumors.^26,27^ This cell line is well-documented for its high in vivo proliferative capacity. In the present study, it successfully promoted robust and progressive tumor development in the hind paw of mice.^11,26,27^

### Thermographic Analysis

Thermographic analysis provides a noninvasive means of evaluating localized hyperthermia, often reflective of underlying inflammatory or hypermetabolic processes within tissues affected by infection, injury, or neoplastic progression.^28–30^ As shown in Figure 2, thermographic images with infrared profiles of representative left paws from animals in each group, while Table 1 presents the comparison of mean temperatures by group for the whole animal, the left paws (gradient), and the tumor.

**Figure 2:**
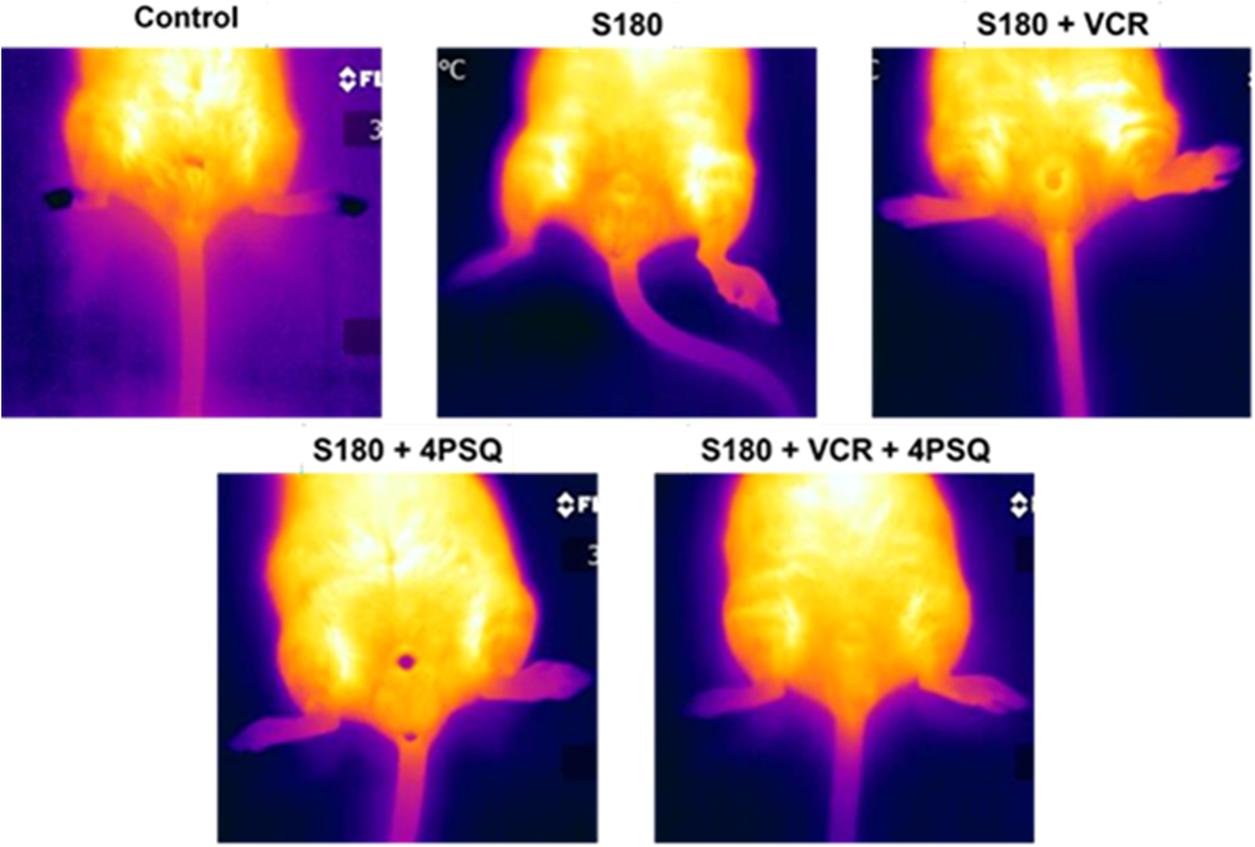
Thermographic images employed to measure tumor temperature (°C).

**Table 1:** Comparison of Mean Temperatures (°C) of the Whole Animal, Left Paws, and Tumors for Each Group (mean ± SD)^b^

group	whole animal	whole paw	tumor^a^
Control	33.2 ± 0.4^A^	23.4 ± 1.5^b^	23.2 ± 0.7^c^
S180	33.0 ± 0.7^A^	28.3 ± 2.2^a^	28.2 ± 1.6^ab^
S180 + VCR	33.8 ± 0.4^A^	28.1 ± 1.8^a^	29.8 ± 3.3^a^
S180 + 4PSQ	32.7 ± 0.7^A^	27.3 ± 1.8^a^	27.1 ± 2.0^ab^
S180 + VCR + 4PSQ	31.7 ± 0.7^A^	26.0 ± 1.2^a.b^	25.4 ± 0.9^bc^

a Central point measurement of the left paw.

b ^A,a,b,c^Superscript letters indicate statistical similarity or difference (One-Way ANOVA, test of Tukey, 95% confidence level).

Following statistical analysis of the results presented in Table 1, no significant differences in mean whole-animal temperatures were observed across groups. However, this pattern was not observed for the mean paw and tumor temperatures. For both whole-paw and tumor measurements, a significant difference was observed between the control group and the other tumor-bearing groups, except for the “S180 + VCR + 4PSQ” group, which consisted of tumor-bearing animals receiving both the chemotherapeutic agent and the test drug.

Regarding measurements of the whole paw, the “S180 + VCR + 4PSQ” group showed temperatures statistically similar to those of the control group, but not significantly different from those of the other tumor-bearing groups. For point temperature measurements in tumors, this same group also reported results that were statistically similar to those of the control group and significantly different from those of the “S180 + VCR” group. Additionally, no significant differences were observed between the “S180 + VCR + 4PSQ” group and the “S180” or “S180 + 4PSQ” groups, likely due to a trend of slightly lower tumor site temperatures in the latter two groups compared to the chemotherapeutic-treated group. These findings may suggest that while the chemotherapeutic agent increases tumor site temperature, the test drug could contribute to its reduction.

In other words, we verified that thermographic imaging revealed a marked increase in surface temperature of the left hind paw, the site of S180 cell inoculation, in all tumor-bearing groups, consistent with tumor-induced inflammation and metabolic upregulation. Notably, animals in the S180 + VCR group exhibited the highest recorded temperature at the tumor site, suggesting that VCR potentiated local inflammatory processes. This observation is supported by the well-established proinflammatory properties of VCR, including enhanced immune activation and cytokine release, as well as increased macrophage release for phagocytic processes, potentially leading to increased peripheral vasodilation and tissue perfusion.^31–33^ The temperature elevation in this group, which exceeded that observed in the S180 group, reinforces the notion that chemotherapeutic agents, while cytotoxic, may exacerbate inflammation and contribute to peripheral nociceptive sensitization. Interestingly, treatment with 4-PSQ alone or in combination with VCR significantly mitigated the thermal response at the tumor site, suggesting anti-inflammatory and/or antitumoral properties that can modulate local thermogenesis. Notably, the thermal profile of the entire body and contralateral limbs remained relatively unchanged across groups, highlighting the localized nature of the thermographic alterations observed. These findings underscore the utility of infrared thermography as a sensitive, noninvasive technique for detecting tumor-associated inflammation and monitoring therapeutic outcomes in preclinical cancer pain models.

Indeed, thermographic technology has been applied in a wide range of biomedical contexts, including breast cancer screening, burn and wound assessment, facial dermatological disorders, febrile condition monitoring, and evaluation of inflammatory joint diseases such as hand osteoarthritis and rheumatoid arthritis.^34^ Notably, our study is among the first to employ this approach to visualize temperature changes in solid tumors induced by S180 cells and to demonstrate its sensitivity to VCR-mediated chemotherapeutic modulation, thus opening new avenues for translational research in oncology and pain assessment.

### Behavior Tests

Cancer pain results from a complex interaction among tumor-induced tissue damage, inflammatory responses, and peripheral or central sensitization, often coexisting with neuropathic mechanisms, especially in the context of chemotherapy regimens such as VCR.^35,36^ In our study, behavioral analyses demonstrated that intraplantar inoculation of S180 cells was sufficient to induce persistent mechanical and thermal hyperalgesia, consistent with a model of inflammatory and tumor-related pain. Notably, coadministration of VCR worsened both types of nociception, particularly during the early stages of the protocol, indicating potentiation of peripheral neuropathy, consistent with VCR’s known neurotoxic profile, which involves microtubule disruption and axonal degeneration. These findings suggest a cumulative nociceptive burden resulting from the combined effects of tumor growth and chemotherapeutic toxicity, closely related to the cancer pain experienced by patients with cancer.^4,37,38^

### Assessment of Mechanical Nociceptive Threshold

The intraplantar inoculation of S180 cells induced a pronounced mechanical hyperalgesia at all assessed time points, day 7 (45%), day 11 (40%), and day 17 (43%), when compared to baseline values (Figure 3A). The group receiving S180 in combination with VCR (S180 + VCR) exhibited an exacerbation of mechanical hypersensitivity relative to the S180 group, with increased nociceptive responses observed on day 7 (27%), day 11 (34%), and day 17 (40%). Treatments with either VCR or 4-PSQ alone, or their combined administration, did not fully restore mechanical sensitivity to control levels. However, beginning on day 11, animals treated with 4-PSQ, whether in the S180 + 4-PSQ or S180 + VCR + 4-PSQ groups, exhibited a significant reduction in mechanical hyperalgesia, indicating a potential antinociceptive effect of 4-PSQ in both tumor- and chemotherapy-associated pain conditions.

**Figure 3:**
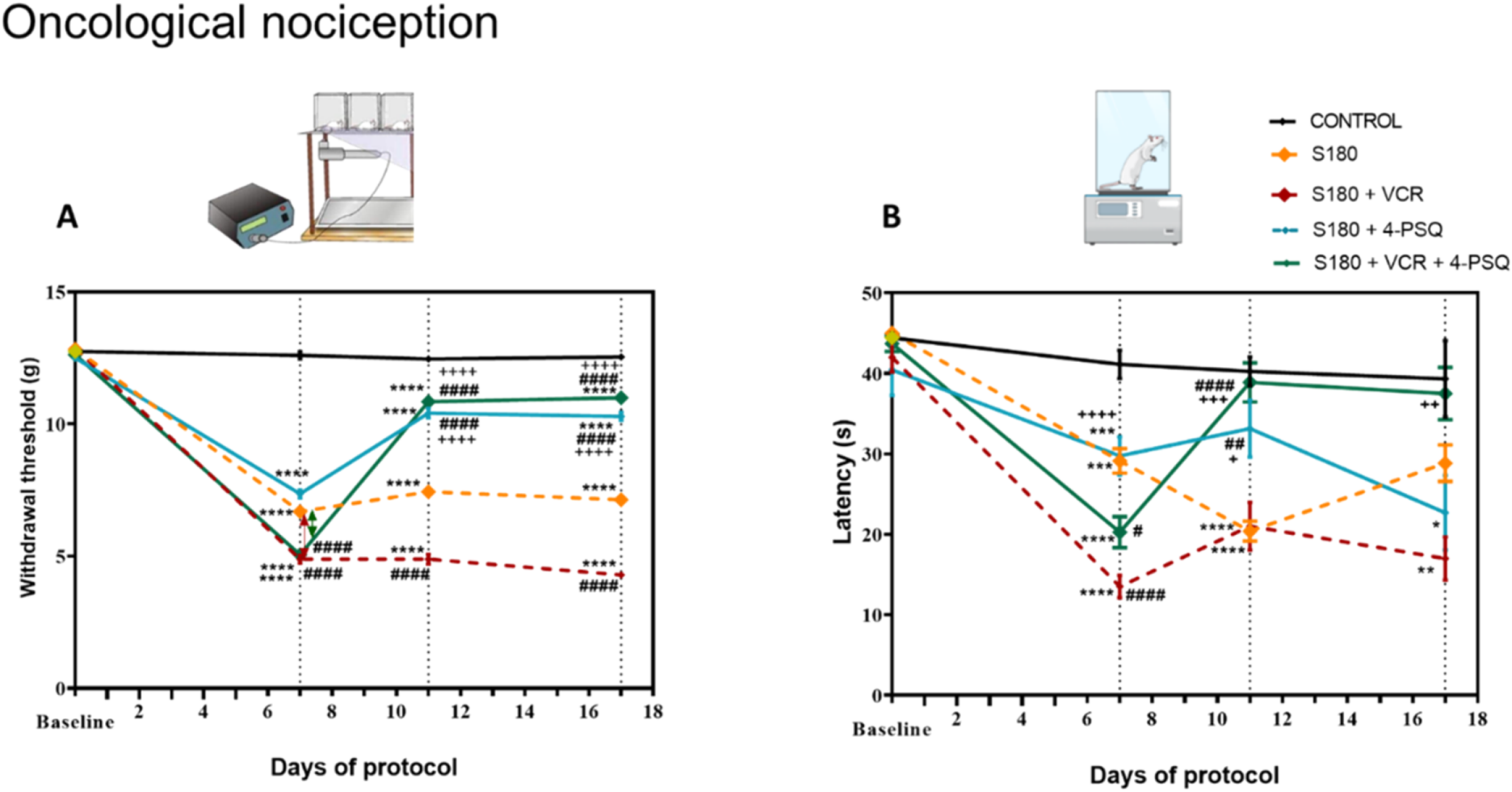
Evaluation of the therapeutic potential of 7-chloro-4-(phenylselanyl)­quinoline (4-PSQ) against mechanical and thermal hyperalgesia in a model of cancer pain induced by Sarcoma (S180 cells) and vincristine (VCR). Evaluation of mechanical hyperalgesia (A) and evaluation of thermal hyperalgesia to heat (B). Each column represents the mean ± standard error of the mean (S.E.M) of 7 animals per group. (*) *P* < 0.05, (**) *P* < 0.01, (***) *P* < 0.001 and (****) *P* < 0.0001 denote significance levels when compared with the control group. (^#^) *P* < 0.05, (^##^) *P* < 0.01 and (^####^) *P* < 0.0001 denote significance levels when compared with the S180 group. (^+^) *P* < 0.05, (^++^) *P* < 0.01, (^+++^) *P* < 0.001, and (^++++^) *P* < 0.0001 denote significance levels compared with the S180 + VCR group. One-way ANOVA followed by Tukey’s test was used.

Notably, 4-PSQ significantly reduced nociceptive behaviors, with effects most pronounced at later time points, in both tumor-bearing mice and those receiving S180 + VCR. This temporal profile suggests mechanisms that extend beyond anti-inflammatory actions, aligning with modulation of redox-sensitive signaling and neuromodulatory pathways reported for organochalcogens.^19,39,40^ Consistent with this mechanistic plausibility, 4-PSQ has demonstrated efficacy across chemotherapy-induced neuropathy models, including those induced by oxaliplatin, paclitaxel, and VCR.^15–18^ Taken together, these findings position 4-PSQ as a promising candidate to mitigate cancer pain arising from both disease progression and neurotoxic treatment.

### Assessment of Thermal Hyperalgesia

Intraplantar inoculation of S180 cells induced a marked increase in thermal sensitivity at all evaluated time points, day 7 (29%), day 11 (49%), and day 17 (27%), compared to the control group (Figure 3B). Co-administration of VCR with S180 (S180 + VCR group) further exacerbated thermal hyperalgesia, with a significant increase in nociceptive responses observed on day 7 (54%) relative to the S180 group.

Treatment with 4-PSQ (S180 + 4-PSQ) effectively restored thermal sensitivity to control levels on days 11 and 17. Furthermore, when 4-PSQ was combined with VCR and tumor induction (S180 + VCR + 4-PSQ group), it significantly reversed the increased thermal hyperalgesia caused by the combined effects of VCR and S180. Notably, the combination treatment showed a longer-lasting antinociceptive effect, with a marked decrease in thermal hypersensitivity maintained between days 11 and 17, exceeding the effectiveness observed in the S180 + 4-PSQ group.

It is important to note that a key inflection point in the thermal sensitivity curve was observed on day 11. At this time point, animals subjected to either S180 inoculation or the combination of S180 and VCR (S180 + VCR) showed similar levels of thermal hyperalgesia. However, by day 17, a divergence in pain responses became clear: the S180 group showed a partial reduction in thermal sensitivity, whereas the S180 + VCR group exhibited a persistent, and even increased, hyperalgesic response. This shift likely indicates the development of a chronic pain state driven by the combined effects of tumor growth and VCR-related neurotoxicity. The continued and heightened nociceptive signaling in the S180 + VCR group may suggest maladaptive plasticity, possibly involving peripheral nociceptor sensitization and/or activation of central pain pathways, including glial activation and the release of proinflammatory cytokines within the spinal cord.^41,42^ These findings highlight the importance of this model in replicating mixed cancer-chemotherapy pain syndromes and emphasize the critical transition between the subacute and chronic phases of nociceptive regulation.

The analgesic profile of 4-PSQ is consistent with a neuroprotective mechanism. Prior studies show that 4-PSQ dampens neuroinflammation by lowering pro-inflammatory cytokines and modulates ATPases (Na^+^/K^+^-, Ca^2+^-, Mg^2+^-ATPases) that govern neuronal excitability, thereby limiting nociceptive transmission along ascending pathways and engaging descending modulatory circuits.^14,16–18,39,43^ In line with this mechanistic plausibility, we observed reversal of thermal and mechanical hypersensitivity, even under chemotherapy-exacerbated conditions, highlighting the relevance of 4-PSQ for mixed cancer pain states. Moreover, this study provides initial evidence that combining 4-PSQ with VCR can aid tumor control while achieving meaningful analgesia, supporting 4-PSQ as a candidate adjunct for cancer pain management. Further study should refine dosing and scheduling to maximize antineoplastic efficacy while preserving the analgesic benefit.

### Ex Vivo Analysis

#### Primary Culture of Peritoneal Macrophages

Peritoneal macrophages were analyzed as indicators of systemic immunoadaptive responses, given their proximity to the intraperitoneal site of VCR administration and their relevance in the context of tumor-induced inflammatory pain.

#### Cytotoxicity

To investigate the immunological impact of tumor progression and therapeutic interventions, cytotoxicity and proliferation assays were performed on peritoneal macrophages collected at the end point of the experimental protocol (Figure 4).

**Figure 4:**
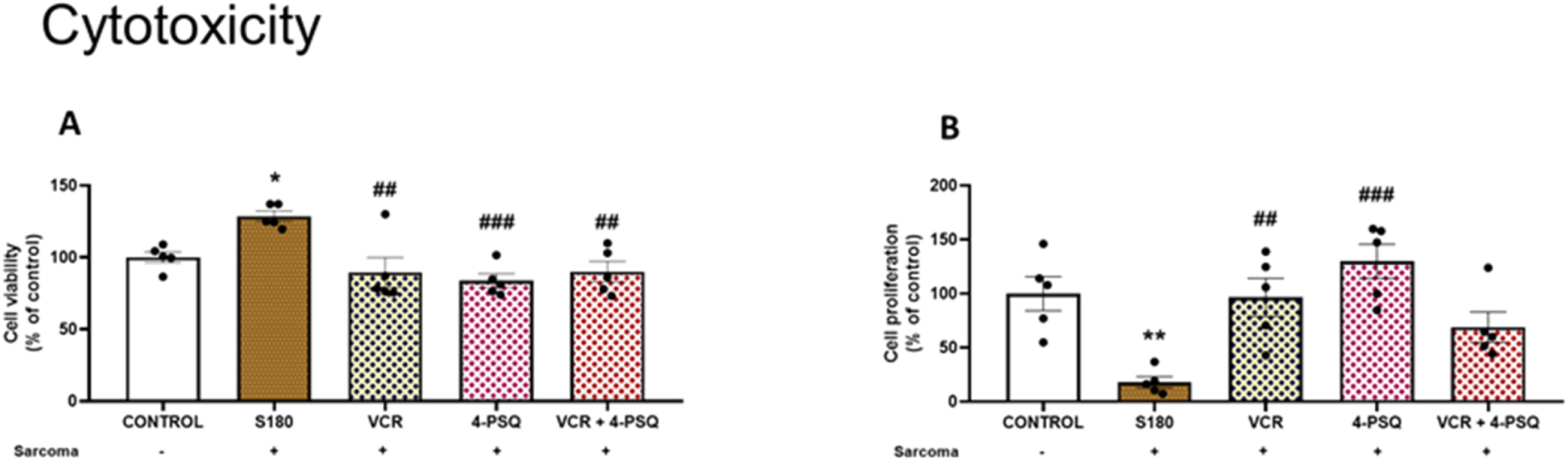
Evaluation of 7-chloro-4-(phenylselanyl)­quinoline (4-PSQ), Sarcoma (S180 cells), and vincristine (VCR) on the cytotoxicity of macrophages from peritoneal cavity fluids. Analysis of cell viability (A) and cell proliferation (B). Each column represents the mean ± standard error of the mean (S.E.M.) of 5–6 animals per group. (*) *P* < 0.05 and (**) *P* < 0.01 denote significance levels when compared with the control group. (^##^) *P* < 0.01 and (^###^) *P* < 0.001 denote significance levels when compared with the S180 group. One-way ANOVA followed by Tukey’s test was used.

As shown in Figure 4A, the MTT assay revealed that inoculation with S180 cells resulted in a significant increase in macrophage viability (25%) compared to the control group, indicating heightened metabolic activity. Treatments with VCR, 4-PSQ, or their combination (VCR + 4-PSQ) effectively restored macrophage viability to control levels.

To further evaluate the functional state of these immune cells under tumor-induced inflammatory conditions, cell proliferation was assessed using the SRB assay (Figure 4B). Contrary to the viability results, S180 inoculation significantly suppressed macrophage proliferation (82%), indicating a potential immunosuppressive or cell cycle-arresting effect of the tumor microenvironment. Both VCR and 4-PSQ, when given alone, counteracted this suppression and restored proliferation to near baseline levels. However, the combined therapy (VCR + 4-PSQ) did not reverse the inhibitory effect on proliferation, suggesting a possible interference or antagonistic interaction in the context of tumor-driven immunomodulation.

The analysis of peritoneal macrophages revealed functional dysregulation following inoculation with S180 cells. Although cell viability increased (MTT assay), proliferation was suppressed (SRB assay), indicating a metabolically active but nonproliferative profile, which is consistent with features commonly described in tumor-associated macrophages (TAMs). This functional pattern may reflect an immunomodulatory macrophage profile commonly described in tumor-associated environments, which is typically associated with immunomodulatory activity rather than proliferative expansion. However, it is important to note that no direct phenotypic markers of macrophage polarization (e.g., CD206, Arg-1, or cytokine profiling) were evaluated in the present study, and therefore, this interpretation should be considered inferential rather than definitive.^44,45^

Considering that the peritoneal cavity typically contains few resident macrophages, the increased cell number likely reflects monocyte recruitment from the bone marrow, consistent with previous findings.^31^ These data support the notion that tumor burden and chemotherapeutic stress trigger systemic immune mobilization.

VCR, known for its neurotoxic and proinflammatory effects via microtubule disruption,^45,46^ surprisingly did not intensify the inflammatory phenotype of peritoneal macrophages when coadministered with S180 cells. This may be attributed to tissue-specific pharmacokinetics or the relatively immunologically quiescent nature of the peritoneal environment. Moreover, VCR’s cytostatic action could restrict immune cell expansion without inducing a robust inflammatory response, particularly outside directly affected tissues.^47,48^ Co-treatment with VCR and 4-PSQ restored both macrophage viability and proliferative capacity, indicating therapeutic modulation of tumor-induced immune dysfunction. These results highlight a potential immunoregulatory effect of 4-PSQ, particularly relevant in the context of cancer pain and chemotherapy-induced neuroinflammation, where macrophage dynamics play a central role in disease progression and resolution.

#### Oxidative Stress Analysis

Parallel to functional changes, S180 inoculation also disrupted redox balance in peritoneal macrophages, evidenced by decreased ROS, SOD, CAT, and nonprotein thiol levels, features that have been associated with immunoregulatory macrophage profiles in tumor contexts.^49,50^

As shown in Figure 5A, inoculation with S180 cells caused a significant decrease of about 73% in intracellular ROS levels in peritoneal macrophages compared to controls. Neither VCR administration nor 4-PSQ treatment, alone or in combination, significantly affected ROS production.

**Figure 5:**
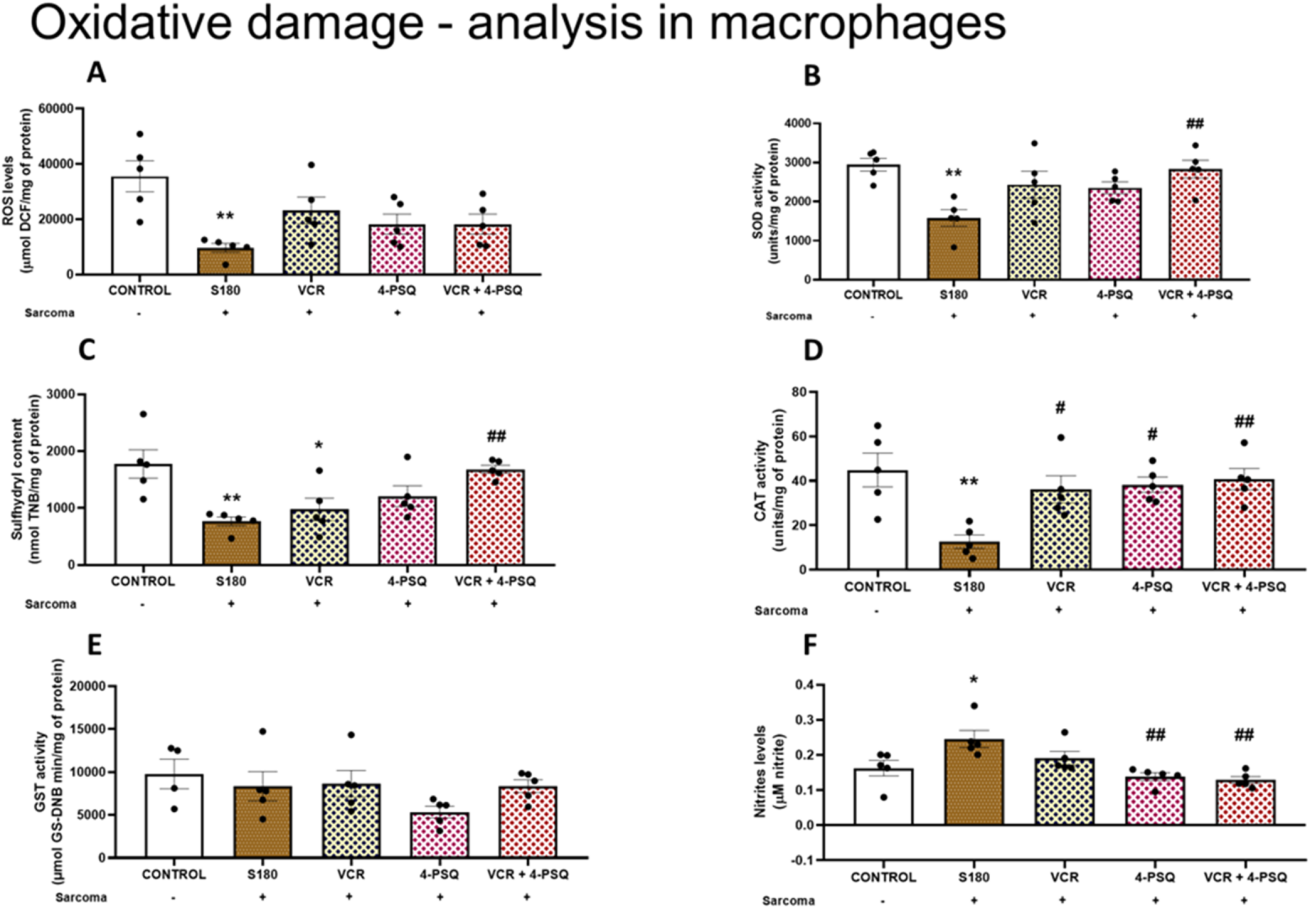
Evaluation of 7-chloro-4-(phenylselanyl)­quinoline (4-PSQ), Sarcoma (S180 cells), and vincristine (VCR) on oxidative damage of peritoneal cavity fluid macrophages. Analysis of reactive oxygen species (ROS) (A); superoxide dismutase (SOD) activity (B); sulfhydryl content (C); catalase (CAT) activity (D); glutathione S-transferase (GST) activity (E); and nitrite levels (F). Each column represents the mean ± standard error of the mean (S.E.M.) of 5–6 animals per group. (*) *P* < 0.05 and (**) *P* < 0.01 denote significance levels when compared with the control group. (^#^) *P* < 0.05 and (^##^) *P* < 0.01 denote significance levels when compared with the S180 group. One-way ANOVA followed by Tukey’s test was used.

SOD activity was markedly inhibited following S180 inoculation, showing a 46% decrease compared with the control group (Figure 5B). Notably, combined treatment with VCR and 4-PSQ restored SOD activity to baseline levels. Regarding the sulfhydryl content, tumor inoculation induced a substantial depletion (∼57%) (Figure 5C). VCR treatment alone and the VCR + 4-PSQ combination effectively reversed this depletion, restoring sulfhydryl levels in peritoneal macrophages.

CAT activity, another key antioxidant defense enzyme, was inhibited (72%) in the S180 group compared to controls (Figure 5D). Both individual and combined treatments restored CAT activity. GST activity, which is involved in xenobiotic detoxification, remained unchanged across all experimental groups, indicating no significant modulation by tumor presence or therapeutic interventions (Figure 5E).

Nitrite levels, which indicate oxidative and inflammatory processes, increased by 51% after S180 inoculation (Figure 5F). Treatment with 4-PSQ, either alone or combined with VCR, effectively brought nitrite concentrations in peritoneal macrophages back to normal.

This redox suppression likely reflects tumor-mediated metabolic reprogramming through interleukin-10 (IL-10) and transforming growth factor (TGF-β) signaling.^50,51^ VCR alone partially reversed this effect, while VCR + 4-PSQ fully restored redox parameters, suggesting a synergistic action on the antioxidant machinery. Interestingly, although ROS levels remained low, nitrite accumulation indicated enhanced nitrosative stress, likely mediated by iNOS activity, suggesting a tumor-driven reprogramming of macrophage oxidative and nitrosative pathways. These findings indicate a complex alteration in macrophage redox pathways, characterized by increased nitrosative activity alongside suppressed antioxidant defenses (SOD and CAT). Notably, 4-PSQ, alone or in combination with VCR, restored sulfhydryl and nitrite levels, suggesting its potential to regulate macrophage redox status and counteract tumor-induced immune dysfunction.

The lack of significant changes in GST activity in peritoneal macrophages exposed to S180, VCR, and 4-PSQ suggests that phase II detoxification is preserved under these conditions. This may reflect insufficient electrophilic stress to induce GST upregulation or the preferential activation of other antioxidant pathways, such as CAT or SOD. Additionally, GST may not be the primary detoxification route for reactive species generated, or temporal dynamics may have been missed. These results highlight the complexity of macrophage antioxidant responses in the context of tumors and chemotherapy. In summary, the results indicate a heterogeneous and context-dependent macrophage activation state, rather than a clear polarization into classical M1 or M2 phenotypes, increased nitrosylation associated with inflammation, and inhibited CAT and SOD activities following tumor inoculation. In contrast, combined 4-PSQ and VCR treatment suggests a potential role in modulating macrophage functional alterations associated with tumor-induced immune dysregulation in this Sarcoma model.

#### Histopathological Evaluation

Histological analysis of the tarsal region in control animals showed preserved tissue architecture, with no neoplastic infiltration or muscle damage (Figure 6; Slides 1–3). S180-inoculated mice exhibited extensive proliferation of pleomorphic tumor cells invading subcutaneous tissue, bone, and skeletal muscle, with dense neutrophilic infiltrates, cellular debris, frequent mitoses, and severe muscle necrosis (+++) (Figure 6; Slides 4–6).^11,52^ These findings reflect a highly aggressive tumor phenotype, with inflammation likely contributing both to tissue remodeling and tumor progression.

**Figure 6:**
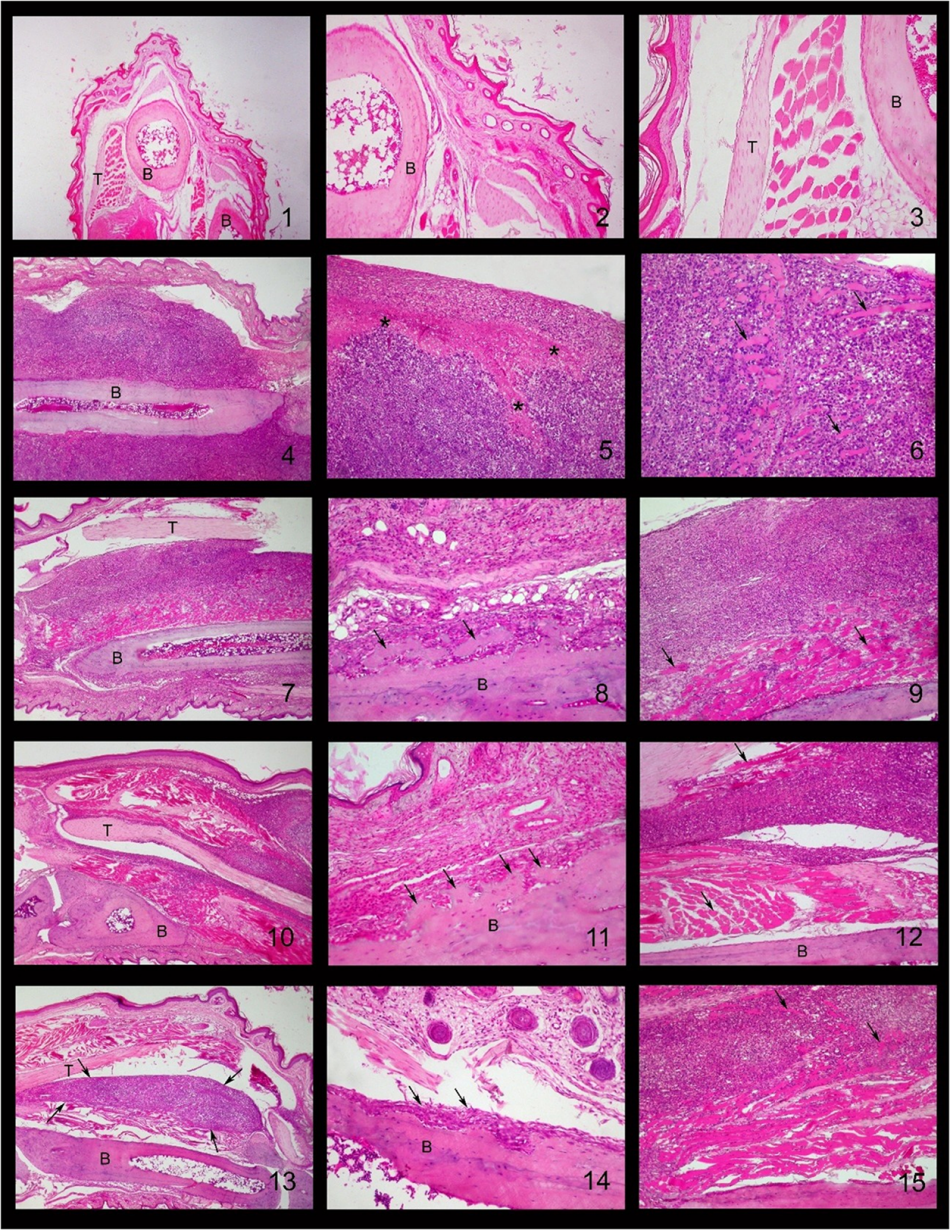
Histopathology of the tarsal (paw) region after S180 inoculation (i.pl.) and treatment with 7-chloro-4-(phenylselanyl)­quinoline (4-PSQ; 1 mg/kg, p.o.) and/or vincristine (VCR; 0.1 mg/kg, i.p.). Hematoxylin–eosin (H&E). Labels: **B** = bone; **T** = tendon; * = necrotic debris; **arrows** indicate features described. **Slides 1–3** (controls, no sarcoma): preserved architecture of T and B (×40); intact connective tissue with well-preserved T/B (×100); normal skeletal muscle and B without neoplastic infiltration (×200). **Slides 4–6** (S180, untreated): extensive neoplastic proliferation infiltrating subcutis and encircling B (×40); abundant * within tumor mass (×100); widespread skeletal muscle destruction with residual fibers (arrow) amid dense neoplastic infiltration (×200). **Slides 7–9** (S180+VCR): marked reduction in tumor burden with preserved B and T (×40); focal bone degradation with neoplastic invasion of bone matrix and discrete bone fragments (arrow), B indicated (×200); partial preservation of muscle architecture (arrow) and attenuated invasion vs untreated, B shown (×100). **Slides 10–12** (S180 + 4-PSQ): substantial decrease in tumor cell density and lesion area vs untreated and VCR groups, B/T evident (×40); neoplastic infiltration into bone persists, though less severe than with VCR monotherapy; bone undergoing resorption (arrow), B indicated (×200); decreased tumor infiltration and preserved muscle fibers (arrow) with less destruction than VCR, B shown (×100). **Slides 13–15** (S180+VCR + 4-PSQ): spatially confined tumor regions (between arrows) with improved local control vs other arms; preserved B/T (×40); focal bone invasion persists but is markedly attenuated; tumor cells invading bone (arrow), B indicated (×200); residual neoplastic infiltration interspersed with skeletal muscle and inflammatory infiltrates (arrow), lower than in VCR or vehicle groups (×100).

VCR treatment partially reduced tumor area and subcutaneous invasion (Figure 6; Slides 7–12), leading to less muscle destruction and a milder inflammatory response (muscle damage ++), although bone involvement and necrosis persisted.^53,54^ Furthermore, the sustained muscle necrosis and inflammation observed align with clinical and experimental evidence of VCR-induced tissue toxicity, including neurotoxicity and inflammatory side effects, which may exacerbate local tissue damage despite its antitumor effects.^55–57^

Treatment with 4-PSQ attenuated tumor infiltration, preserved muscle tissue, and reduced inflammation (muscle damage ++), suggesting protective effects through antioxidant and anti-inflammatory mechanisms. Combination therapy (VCR + 4-PSQ) yielded the most favorable outcomes, confining neoplastic cells, minimizing tissue destruction, attenuating bone involvement, and producing only mild muscle necrosis and inflammation (muscle damage +) (Figure 6; Slides 13–15). These results indicate that VCR provides direct cytotoxicity while 4-PSQ modulates oxidative stress and inflammatory pathways, supporting its potential as an adjuvant to enhance tissue preservation and antitumor efficacy.

### Biochemical Assays

#### TBARS Levels

Given the extensive tissue injury and inflammation observed during S180 tumor progression and VCR treatment, we assessed oxidative damage via TBARS quantification in CNS tissues. In the cerebral cortex, S180 and S180 + VCR groups showed significantly increased TBARS (45% and 38% vs control), which were restored to baseline by 4-PSQ, alone or combined with VCR (Figure 7A). In the spinal cord, TBARS increased 87% (S180) and 71% (VCR), also reversed by 4-PSQ (Figure 7B). In the hippocampus and cerebellum, TBARS showed nonsignificant upward trends in S180 mice, but 4-PSQ significantly reduced levels, demonstrating antioxidant effects even under subclinical oxidative stress (Figure 7C,D).

**Figure 7:**
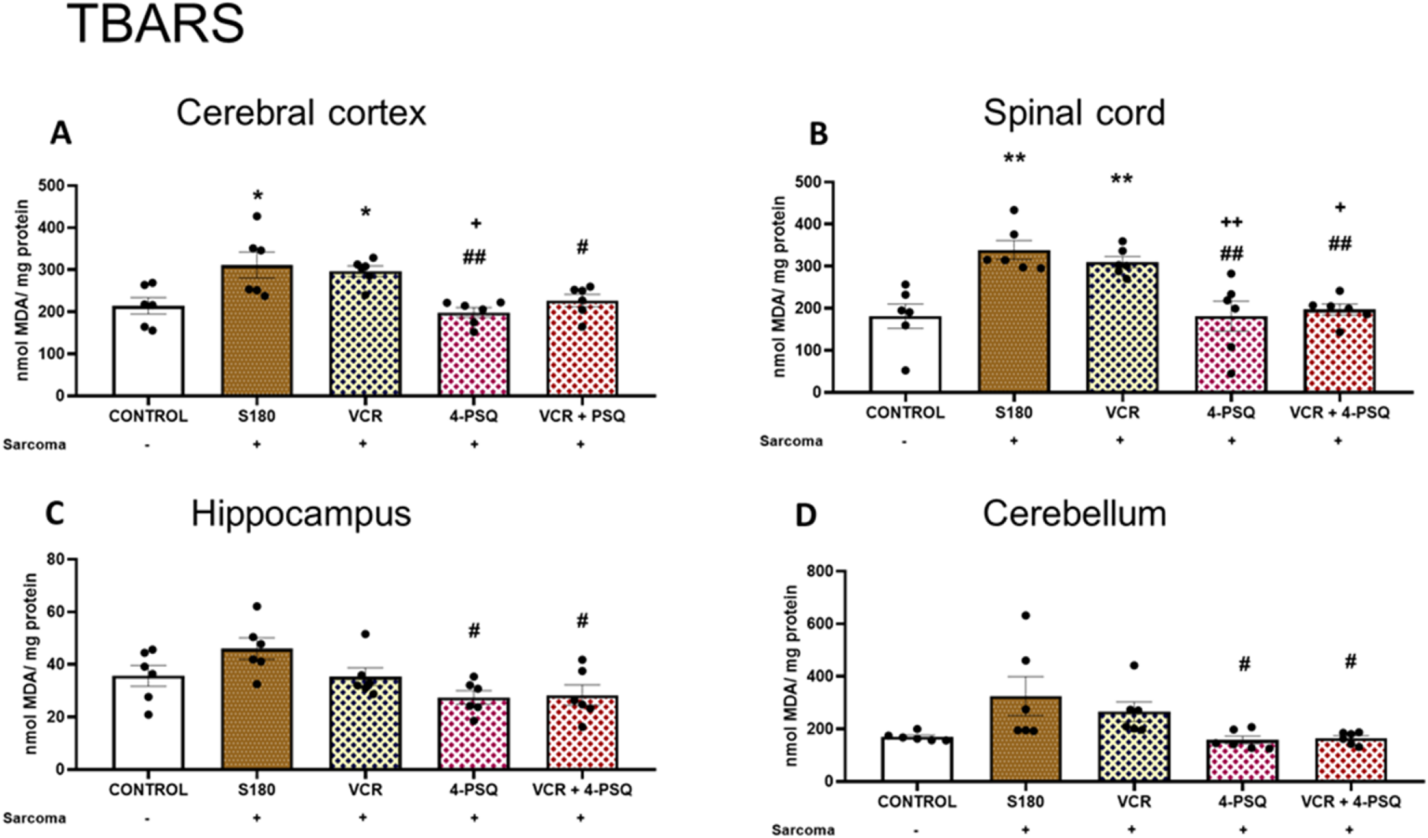
Evaluation of 7-chloro-4-(phenylselanyl)­quinoline (4-PSQ), Sarcoma (S180 cells), and vincristine (VCR) in the thiobarbituric acid reactive species (TBARS) assay. Lipid peroxidation analysis by TBARS in the cerebral cortex (A), spinal cord (B), hippocampus (C), and cerebellum (D) of mice. Each column represents the mean ± standard error of the mean (S.E.M) of 6–7 animals per group. (*) *P* < 0.05 and (**) *P* < 0.01 denote significance levels when compared with the control group. (^#^) *P* < 0.05 and (^##^) *P* < 0.01 denote significance levels compared with the S180 group. (^+^) *P* < 0.05 and (^++^) *P* < 0.01 denote significance levels compared with the S180 + VCR group. One-way ANOVA followed by Tukey’s test was used.

These findings indicate that the tumor microenvironment and VCR promote lipid peroxidation and metabolic dysfunction, contributing to CNS neurotoxicity and potentially to peripheral neuropathy.^58,59^ The cerebral cortex and spinal cord are key for pain modulation^60,61^ and oxidative stress here may enhance hyperalgesia. 4-PSQ likely exerts neuroprotection through its lipophilic properties, stabilizing membranes and attenuating reactive species, thereby preserving neuronal integrity in both overt and subclinical oxidative conditions.

#### Na^+^ K^+^-ATPase and Mg^2+^-ATPase Activities

Na^+^,K^+^-ATPase, a membrane-bound enzyme essential for neuronal excitability, is highly susceptible to lipid peroxidation.^17,62,63^ In the cerebral cortex, S180 inoculation significantly increased Na^+^,K^+^-ATPase activity (106%), likely reflecting compensatory neuroexcitation and inflammation, while VCR normalized this hyperactivity.^64^ VCR treatment normalized this cortical hyperactivity, possibly through oxidative stress or post-translational modification of the β-subunit,^65^ which can promote Na^+^ accumulation, impair Na^+^/Ca^2+^ exchange, and precipitate Ca^2+^ overload, contributing to excitotoxicity and apoptosis.^66^ Interestingly, 4-PSQ alone maintained elevated cortical activity, suggesting adaptive modulation of ion homeostasis under stress conditions. In the hippocampus, VCR reduced Na^+^,K^+^-ATPase activity, which 4-PSQ did not restore, highlighting regional vulnerability. In the spinal cord and cerebellum, Na^+^,K^+^-ATPase activity remained stable, with trends toward elevation in some groups, indicating region-specific responses to tumor- and chemotherapy-induced stress (Figure 8A–D).

**Figure 8:**
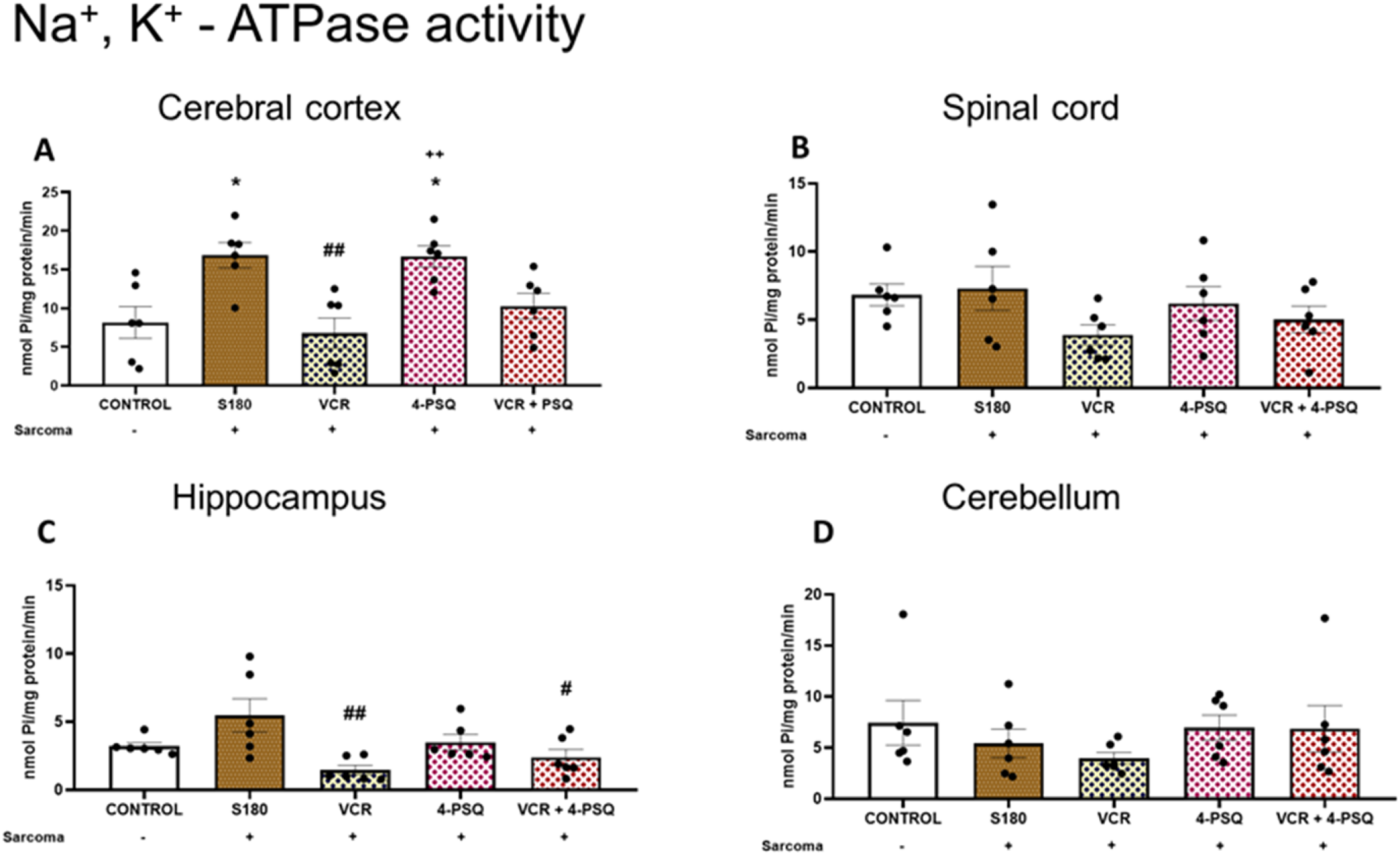
Evaluation of 7-chloro-4-(phenylselanyl)­quinoline (4-PSQ), Sarcoma (S180 cells) and vincristine (VCR) on Na^+^, K^+^-ATPase activity. Enzymatic analysis of Na^+^, K^+^-ATPase in the cerebral cortex (A), spinal cord (B), hippocampus (C), and cerebellum (D). Each column represents the mean ± standard error of the mean (S.E.M.) of 6–7 animals per group. (*) *P* < 0.05 indicates significance compared with the control group. (^#^) *P* < 0.05 and (^##^) *P* < 0.01 denote significance levels when compared with the S180 group. (^++^) *P* < 0.01 denotes significance levels when compared with the S180 + VCR group. One-way ANOVA followed by Tukey’s test was used.

Mg^2+^-ATPase activity revealed further region-specific bioenergetic alterations. Cortical activity was significantly inhibited only in the S180 + VCR group, reflecting compounded mitochondrial and oxidative stress. In the spinal cord, cotreatment with VCR + 4-PSQ enhanced Mg^2+^-ATPase activity, consistent with protective effects via attenuation of oxidative damage. Hippocampal activity, elevated by S180 and suppressed by VCR, was restored by 4-PSQ, which also normalized cerebellar activity (Figure 9A–D). These findings indicate that ATPase dysregulation is region-specific and linked to tumor- and drug-induced stress. The ability of 4-PSQ to preserve ATPase activity underscores its potential in maintaining CNS bioenergetics and neuronal integrity under pathological conditions.

**Figure 9:**
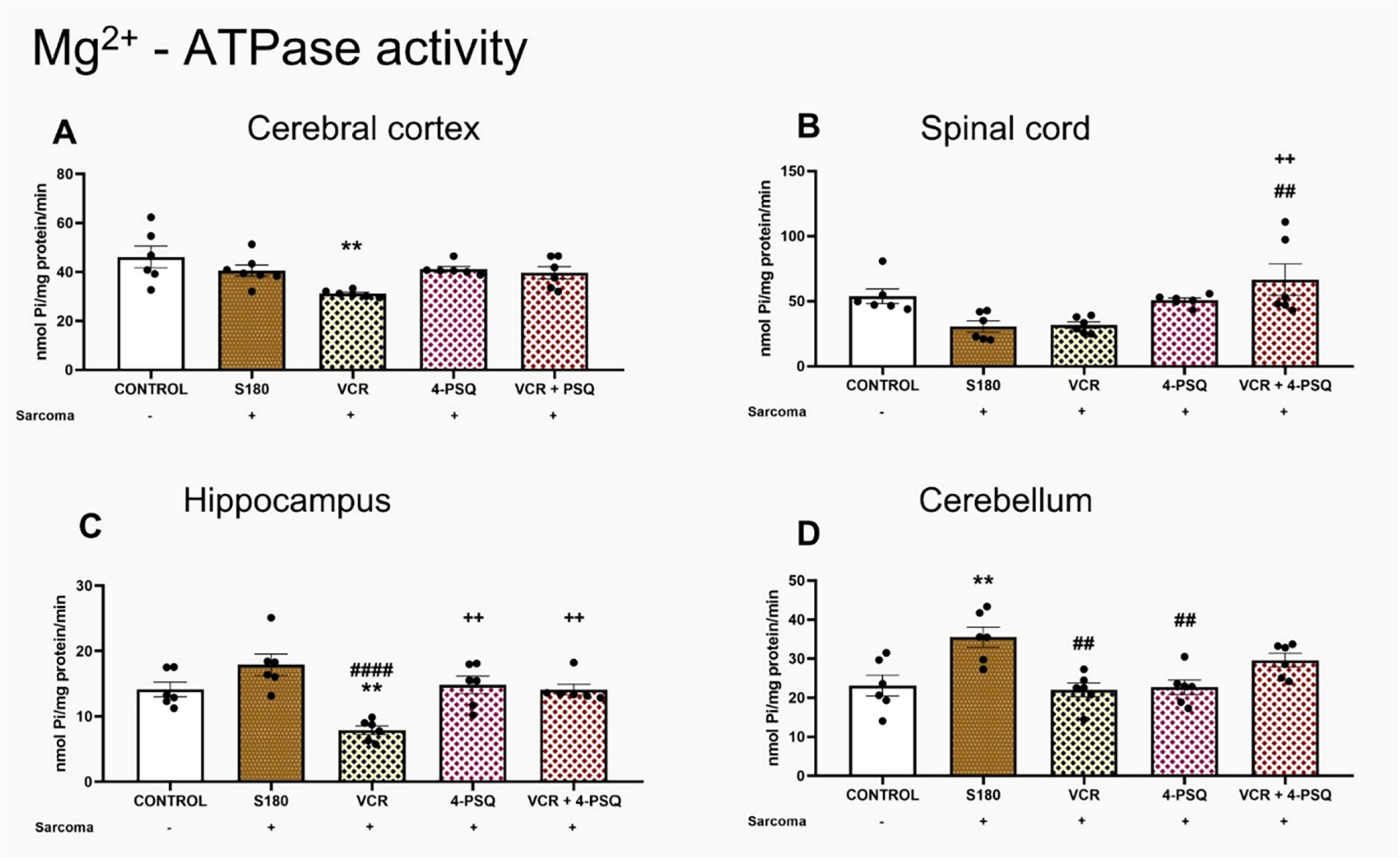
Evaluation of 7-chloro-4-(phenylselanyl)­quinoline (4-PSQ), Sarcoma (S180 cells), and vincristine (VCR) on Mg^2+^-ATPase activity. Enzymatic analysis of Mg^2+^-ATPase in the cerebral cortex (A), spinal cord (B), hippocampus (C), and cerebellum (D). Each column represents the mean ± standard error of the mean (S.E.M.) of 6–7 animals per group. (**) *P* < 0.01 denotes significance levels when compared with the control group. (^##^) *P* < 0.01 and (^####^) *P* < 0.0001 denote significance levels when compared with the S180 group. (^++^) *P* < 0.01 denotes significance levels when compared with the S180 + VCR group. One-way ANOVA followed by Tukey’s test was used.

Taken together, these findings indicate that ATPase activity is modulated in a region-specific manner under tumor- and chemotherapy-induced conditions. Although alterations in ATPase activity have been associated in the literature with neuroinflammation, oxidative stress, and nociceptive processing, the present results should be interpreted as associative rather than mechanistic. Thus, the observed modulation of ATPases may reflect broader biochemical and neuroinflammatory changes related to the experimental conditions, rather than a direct mediator of the analgesic effects of 4-PSQ. Nonetheless, these findings contribute to the understanding of the neurobiological context in which cancer-related and neuropathic pain develop and are modulated.

## Conclusion

This study explored the therapeutic potential of 4-PSQ in a clinically relevant model of cancer pain induced by S180 cells inoculation and VCR. The following key findings underscore the multifaceted effects of 4-PSQ in modulating tumor progression, pain behavior, neuroinflammation, and oxidative imbalance:

- *Analgesic efficacy in mixed pain states*: 4-PSQ effectively reversed mechanical and thermal hyperalgesia, even under VCR-potentiated conditions, indicating efficacy against both inflammatory and neuropathic components of cancer pain;
- *Modulation of tumor progression and edema*: Treatment with 4-PSQ, alone or in combination with VCR, significantly reduced paw swelling and mass, supporting anti-inflammatory and antitumor effects;
- *Immunomodulatory and antioxidant actions*: 4-PSQ restored redox balance and proliferation in peritoneal macrophages, reversing M2-like polarization; normalized ROS levels; regulated of the activity of CAT and SOD enzymes, as well as thiol levels, indicating strong immunoregulatory and antioxidant properties.
- *Neuroprotective preservation of ATPase activity*: The compound preserved Na^+^, K^+^, and Mg^2+^-ATPase activities in CNS regions affected by tumor burden and VCR, supporting energy homeostasis and neuronal integrity.
- *Thermographic evidence of anti-inflammatory action*: Infrared thermography revealed that 4-PSQ attenuated VCR-induced hyperthermia at the tumor site, confirming its capacity to mitigate localized inflammation.

These findings collectively highlight 4-PSQ as a promising adjuvant strategy in the management of cancer pain syndromes, offering neuroprotective, antioxidant, anti-inflammatory, and analgesic effects in a model of tumor progression combined with chemotherapy-induced neuropathy. Future studies should explore its translational potential in broader oncological contexts.

A limitation of the present study is the absence of direct comparisons between 4-PSQ and reference drugs for neuropathic pain, such as pregabalin and duloxetine, which would further strengthen the translational relevance of our findings. This was primarily due to the extensive scope of the current experimental design, which focused on multiple oncological pain components, including tumor-induced and chemotherapy-induced neuropathic pain, as well as ethical constraints on animal use that limited the number of experimental groups. Future studies from our group are underway to directly compare the efficacy of 4-PSQ with reference drug, particularly duloxetine, in similar models.

## Methods

### S180 Sarcoma Mouse Model

S180 cells were obtained from the Rio de Janeiro Cell Bank (APABCAM, Federal University of Rio de Janeiro, Brazil). The cells were cultured in Dulbecco’s Modified Eagle Medium (DMEM) supplemented with 10% fetal bovine serum, 1% penicillin-streptomycin and 1% amphotericin B. Cultures were maintained at 37 °C in a humidified incubator with 5% CO_2_. Cell counts were performed using a Neubauer hemocytometer and the Trypan Blue dye exclusion method. For in vivo experiments, animals were inoculated with 20 μL of a suspension containing 1 × 10^6^ tumor cells.

### Animals

Experiments were conducted on 60-day-old male Swiss mice. The animals were kept under controlled environmental conditions (temperature: 22  ±  2 °C; humidity: 20–80%) and a 12-h light/dark cycle, with free access to standard laboratory food and water. All procedures received approval from the Institutional Animal Care and Use Committee of the Federal University of Pelotas, Brazil (protocol no. CEEA 24440–2019) and were performed in accordance with established ethical guidelines.

### Drugs

The compound 4-PSQ (Figure 10) was synthesized and structurally characterized by the Clean Organic Synthesis Laboratory (LASOL) at the Federal University of Pelotas. Structural elucidation was performed using proton and carbon-13 nuclear magnetic resonance spectroscopy (^1^H and ^13^C NMR). Compound purity was assessed via gas chromatography–mass spectrometry (GC–MS), confirming a purity level of 99.9%.^67,68^ VCR sulfate was obtained from INTAS Pharmaceuticals (Batch No. M2103904) and stored at 4 °C.

**Figure 10:**
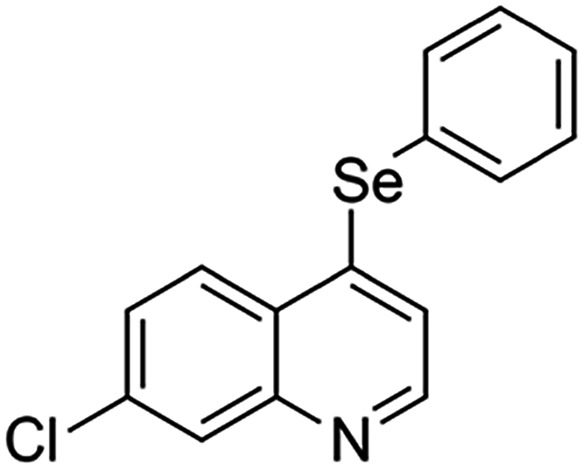
Chemical structure of 7-chloro-4-(phenylselanyl) quinoline (4-PSQ).

The 4-PSQ was prepared at a dose of 1 mg/kg in canola oil (10 mg/mL) and administered orally via gavage (p.o.), considering its lipophilic nature. VCR was prepared at a dose of 0.1 mg/kg in 0.9% saline (10 mg/mL) and administered intraperitoneally (i.p.), following established protocols. Appropriate vehicle controls were included for all experimental conditions. Animals not receiving active treatments were administered the corresponding vehicles following the same route, volume, and schedule, namely canola oil (p.o.) for 4-PSQ and solution saline (i.p.) VCR. All other reagents used for biochemical assays were of high analytical grade and obtained from Sigma-Aldrich (St. Louis, MO, USA).

### Design Experimental for Oncological Pain

Animals were randomly allocated to the experimental groups using a randomization procedure prior to the initiation of treatments. All analyses were conducted following standardized protocols to minimize potential bias. This study aimed to assess cancer pain caused by both VCR and tumor growth using a well-established sarcoma model. Male mice were randomly divided into five experimental groups.

- Group I—Control: received an intraplantar (i.pl.) injection of 25 μL of PBS solution, intraperitoneal (i.p.) injection of 0.9% saline solution (10 mL/kg), and oral administration of canola oil (10 mL/kg).
- Group II—S180: received an injection of 25 μL (i.pl.) of a suspension containing S180 tumor cells, 0.9% saline solution i.p. (10 mL/kg), and oral administration of canola oil (10 mL/kg).
- Group III—S180 + VCR: received S180 cell inoculation (25 μL; i.pl.), VCR was induced at 0.1 mg/kg (i.p.), and oral canola oil (10 mL/kg).
- Group IV—S180 + 4-PSQ: received S180 cell inoculation (25 μL; i.pl.), 0.9% saline i.p. (10 mL/kg), and 4-PSQ treatment at a dose of 1 mg/kg (p.o.).
- Group V—S180 + VCR + 4-PSQ: received S180 cell inoculation (25 μL; i.pl.), VCR at 0.1 mg/kg (i.p.), and 4-PSQ treatment at 1 mg/kg (p.o.).

Baseline nociceptive thresholds were set on day 0. Tumor induction was carried out on day 1 in groups II–V, while group I received only the vehicle. VCR (0.1 mg/kg, intraperitoneally) was administered once daily from days 2 to 6 in groups III and V; the other groups received isotonic saline as a vehicle control. Oral administration of 4-PSQ (1 mg/kg) occurred once daily from days 8 to 17 in groups IV and V, while the remaining groups received the canola oil vehicle (10 mL/kg) orally.

Nociceptive responses, mechanical allodynia, and thermal hyperalgesia were assessed on days 0, 7, 11, and 17. Tumor progression was monitored over time through volumetric measurements of the affected paw every 48 h. On day 18, animals underwent infrared thermographic imaging, followed by euthanasia. Tumor-inoculated tissues were excised for histopathological examination. Central nervous system regions (cerebral cortex, spinal cord, cerebellum, and hippocampus) were harvested for subsequent biochemical analyses, and peritoneal exudates were collected for immunophenotyping of macrophage populations. Figure 11 illustrates the experimental design clearly and structurally, aiding understanding of the temporal sequence and interventions.

**Figure 11:**
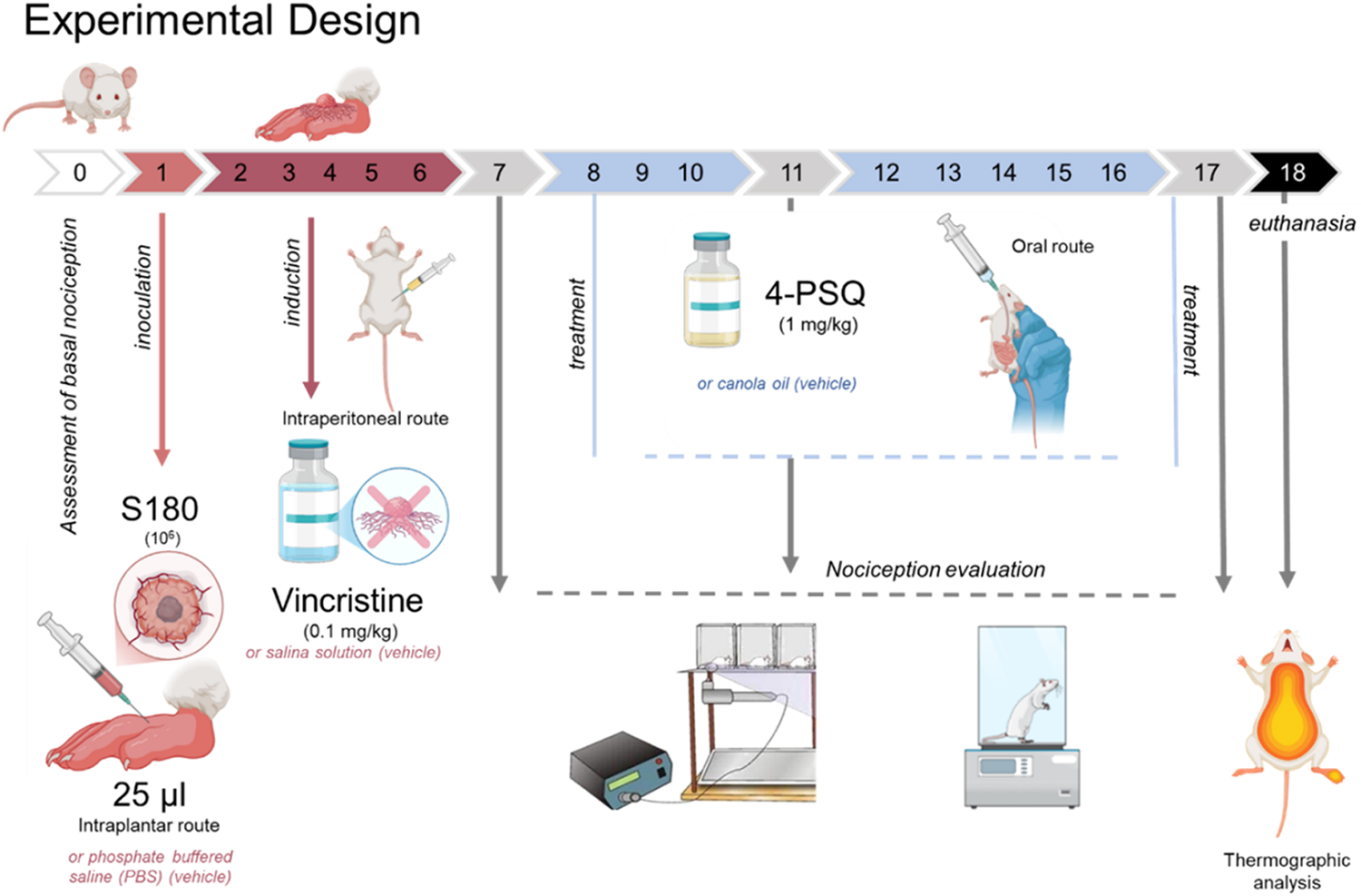
Experimental design scheme. Evaluation of the therapeutic potential of 7-chloro-4-(phenylselanyl)­quinoline (4-PSQ) against cancer pain in Sarcoma (S180 cells) and/or vincristine (VCR)-induced peripheral neuropathy. Male mice were randomly assigned to five experimental groups: (I) Control, (II) S180, (III) S180 + VCR, (IV) S180 + 4-PSQ, and (V) S180 + VCR + 4-PSQ. On day 0, baseline nociceptive thresholds were assessed. On day 1, groups II–V received an intraplantar (i.pl.) injection of 25 μL of a suspension containing 10^6^ S180 tumor cells into the left instep, whereas group I received 25 μL of phosphate buffered saline (PBS) (vehicle, i.pl.). From days 2 to 6, groups III and V were treated with VCR (0.1 mg/kg, intraperitoneally), whereas the remaining groups received 0.9% saline (vehicle, 10 mg/mL, intraperitoneally). From days 8 to 17, animals received daily oral administration of 4-PSQ (1 mg/kg). Nociceptive assessments (mechanical and thermal hyperalgesia) were performed on days 0, 7, 11, and 17. Tumor progression was monitored by measuring paw volume every 2 days. On day 18, thermographic imaging was conducted, followed by euthanasia. Tumor-inoculated paws were harvested for histopathological analysis. Central nervous system tissues (cortex, spinal cord, cerebellum, and hippocampus) were dissected for biochemical assays, and peritoneal exudate was collected for evaluation of macrophage phenotypes.

### Assessment of Tumor Progression

Tumor growth was monitored every 48 h by measuring paw dimensions using a calibrated digital caliper. The S180 Sarcoma cells were previously inoculated into the plantar surface of the left hind paw, and measurements were taken throughout the experimental period to assess tumor progression. Two anatomical parameters were evaluated: *paw width*—defined as the lateral distance across the paw, corresponding to the frontal (coronal) plane, measured from the medial to the lateral margins; and *paw depth*—defined as the dorsoplantar distance, corresponding to the sagittal plane, measured from the plantar (ventral) surface to the dorsal aspect of the paw.

All measurements were taken with the animals gently restrained to reduce stress and movement. Data were recorded in centimeters (cm) and shown as average values for each time point. Progressive increases in paw size were seen as signs of tumor growth and related swelling, enabling a noninvasive, ongoing assessment of tumor burden in live subjects.

### Assessment of Tumor-Associated Edema

To measure tumor-associated edema, a post-mortem analysis was conducted after euthanasia on day 18 of the protocol. The left and right hind paws were surgically removed at the ankle joint immediately after sacrifice and weighed using a precision analytical balance (±0.0001 g accuracy).

Edema was assessed by calculating the difference in tissue mass between the tumor-bearing (left) paw and the contralateral, noninoculated (right) paw of the same animal, serving as an internal control. This method enables individualized normalization and reduces interanimal variability caused by baseline anatomical differences. The following formula was used to estimate the extent of edema

$$\mathrm{Paw}\;\mathrm{Edema}\;\left(\mathrm{mg}\right)={\mathrm{Weight}}_{\mathrm{LeftPaw}\left(\mathrm{tumor}\right)}-{\mathrm{Weight}}_{\mathrm{RightPaw}\left(\mathrm{control}\right)}$$

This mass differential reflects the contribution of both tumor growth and associated inflammatory edema, providing a robust end point for comparative analysis of treatment efficacy.

### Thermographic Analysis

Thermographic imaging was performed using a long-wave infrared camera (7.5–13.0 μm, FLIR E60, FLIR Systems, USA) with a resolution of 320 × 240 pixels and a frame rate of 30 Hz. Images were recorded and processed using ResearchIR software (version 3.5, FLIR Systems). Anesthetized mice were imaged at a distance of 0.15 m in a temperature-controlled room (20 ± 1 °C). Thermal analysis was conducted using three approaches: (i) temperature range (°C) across the entire body, including dorsal and ventral surfaces; (ii) temperature gradient (°C) from the ankle to the tip of the left paw (tumor local); and (iii) point temperature (°C) at the paw center for control animals and at the tumor center for experimental animals.

### Behavior Tests

#### Assessment of Mechanical Nociceptive Threshold

Mechanical sensitivity was evaluated on days 0, 7, 11, and 17 using a modified version of the method originally described by Alamri et al.^69^ To ensure consistency and reduce environmental stress, mice were individually acclimated for at least 30 min in transparent acrylic enclosures placed over an elevated wire mesh platform, providing unobstructed access to the plantar surface of the hind paws. All procedures were performed in a temperature-controlled, noise-free environment.

Mechanical nociceptive thresholds were evaluated using a digital hand-held force transducer (Digital Analgesimeter; Insight, São Paulo, Brazil), equipped with a blunt polypropylene tip. A gradually increasing force was applied perpendicularly to the central region of the plantar surface of the hind paw until a reflexive withdrawal response was elicited. The force required to induce paw withdrawal was automatically recorded in grams (g) and considered the mechanical withdrawal threshold.

For each time point, the mechanical threshold was determined based on ten consecutive measurements per animal, performed in a rotating sequence across groups of up to 12 animals housed simultaneously within the testing apparatus. The procedure involved applying the stimulus to each animal in turn and then repeating the sequence until a total of ten rounds were completed. This design ensured consistent interstimulus intervals, minimizing habituation or sensitization effects. The mean withdrawal threshold from the ten measurements was calculated for each animal and used for subsequent statistical analysis. Data were presented as line graphs depicting the temporal profile of mechanical hypersensitivity across experimental conditions.

#### Assessment of Thermal Hyperalgesia

Thermal nociception was evaluated using a modified version of the hot plate test originally described by Woolfe and MacDonald.^70^ This assay is widely recognized as a reliable behavioral paradigm for assessing supraspinal nociceptive responses in rodents. Evaluations were conducted on experimental days 0, 7, 11, and 17 to monitor the development of thermal hyperalgesia over time.

Mice were individually placed in a transparent acrylic box positioned on a heated metal surface maintained at a constant temperature of 52 ± 1 °C. The latency to the first nociceptive response, characterized by hind paw licking, withdrawal, or jumping, was recorded in seconds and used as an index of thermal sensitivity. To prevent tissue damage, a maximum cutoff time of 45 s was enforced for all animals. Shortened response latencies were interpreted as indicative of increased thermal sensitivity. Each animal was tested only once per session, and all measurements were performed in a quiet, temperature-controlled room to minimize external stressors. Results were expressed as paw withdrawal latency (seconds) and analyzed to determine treatment-induced changes in thermal nociceptive thresholds.

#### Ex Vivo Analysis

On day 18 of the experimental protocol, animals were euthanized, and tissue samples were systematically collected for comprehensive multiparametric analyses. Peritoneal fluid was first harvested to characterize macrophage phenotypes, evaluate cytotoxic functions, and assess oxidative damage profiles. Subsequently, hind paws were excised, weighed to quantify edema, and processed for detailed histopathological evaluation. Additionally, discrete regions of the central nervous system, including the cerebral cortex, spinal cord, cerebellum, and hippocampus, were dissected for biochemical assays measuring ATPase activities (Na^+^/K^+^-ATPase and Mg^2+^-ATPase) and lipid peroxidation marker. These analyses aimed to elucidate biochemical alterations associated with Sarcoma- and VCR-induced oncological pain, as well as to characterize the therapeutic effects of 4-PSQ.

#### Primary Culture of Peritoneal Macrophages

Primary cultures of peritoneal macrophages were established following the protocol described by dos Santos et al.,^71^ with minor modifications. Briefly, macrophages were harvested by lavage of the peritoneal cavity using 5 mL of sterile RPMI 1640 medium devoid of fetal bovine serum (FBS). The recovered cell suspension was centrifuged and washed twice with sterile phosphate-buffered saline (PBS) to remove residual contaminants, followed by resuspension in serum-free RPMI 1640 medium. Cells were then seeded into 6-, 48-, or 96-well culture plates and incubated at 37 °C in a humidified atmosphere containing 5% CO_2_ for 30 min to facilitate selective adherence of macrophages. Subsequently, nonadherent cells were gently removed by washing with serum-free RPMI 1640 medium. The remaining adherent cell population, enriched in peritoneal macrophages, was utilized for downstream functional assays.

### Cytotoxicity

#### Cell Viability Assay

To evaluate cell viability and proliferative capacity, peritoneal macrophages were seeded in 96-well plates at a density of 1 × 10^6^ cells per well and incubated for 18 h under standard culture conditions. Cell metabolic activity was subsequently assessed using the 3-(4,5-dimethylthiazol-2-yl)-2,5-diphenyltetrazolium bromide (MTT) assay, a colorimetric technique based on the enzymatic reduction of the yellow tetrazolium salt MTT to insoluble purple formazan crystals by mitochondrial dehydrogenases in viable cells.

MTT reagent was added to each well at a final concentration of 0.5 mg/mL, and the plates were incubated for 90 min at 37 °C. Following incubation, the supernatant was carefully aspirated, and formazan crystals were solubilized in dimethyl sulfoxide (DMSO) with gentle agitation. Absorbance was quantified at 492 nm using a microplate spectrophotometer (SpectraMax 190, Molecular Devices, San Jose, CA, USA).

Cell viability was calculated relative to untreated controls according to the following formula

$$cell\;\,viability\;rate\;\left(\%\right)=\frac{{OD}_{treated}}{{OD}_{control}}\times 100\%$$

Results were presented as percentage viability relative to control cells.

#### Cell Proliferation Assay

Cell proliferation was evaluated using the sulforhodamine B (SRB) assay, a colorimetric method that quantifies cellular protein content as an indirect measure of cell density and proliferation. The procedure was performed according to the protocol described by Pauwels et al.,^72^ with minor modifications. Following 18 h of treatment, macrophage cultures were gently washed with phosphate-buffered saline (PBS) and fixed by the addition of ice-cold 50% trichloroacetic acid (TCA) for 45 min at 4 °C to precipitate cellular proteins.

After fixation, the plates were thoroughly rinsed five times with distilled water to remove residual TCA. Cells were then stained with 0.4% (w/v) sulforhodamine B solution prepared in 1% acetic acid and incubated at room temperature, protected from light, for 30 min. Excess unbound dye was removed by washing the wells five times with 1% acetic acid. The protein-bound SRB dye was subsequently solubilized in 10 mM Tris base (pH ∼ 10.5), and absorbance was measured at 530 nm using a microplate reader (SpectraMax 190, Molecular Devices, San Jose, CA, USA).

Proliferation rates were expressed as a percentage of the untreated control group according to the formula

$$\mathrm{cell}\;\mathrm{viability}\;\mathrm{rate}\;\left(\%\right)=\frac{{\mathrm{OD}}_{\mathrm{treated}}}{{\mathrm{OD}}_{\mathrm{control}}}\times 100\%$$

#### Preparation of Lysates for Oxidative Stress Assays

After 18 h of incubation, the cultures were washed twice with sterile water, and lysates were prepared manually using a cell scraper. Then, the collected sample was centrifuged at 1000 rpm for 10 min. The pellet was discarded, and the supernatant was used for analysis of oxidative parameter.^73^ Protein determination was carried out according to Lowry et al.,^74^ using bovine serum albumin as a standard.

### Oxidative Stress Analysis

#### Quantification of Reactive Oxygen Species (ROS)

Intracellular ROS levels were measured using the DCFH-DA (2′,7′-dichlorodihydrofluorescein diacetate) assay, following the procedure described by dos Santos et al.,^71^ with slight modifications. Macrophage cultures were incubated with 1 μM DCFH-DA for 30 min at 37 °C in the dark. Upon cellular uptake, DCFH-DA is deacetylated by intracellular esterases and oxidized by ROS to form the fluorescent compound DCF. Fluorescence intensity was then measured using a microplate reader (SpectraMax190) with excitation at 485 nm and emission at 520 nm. ROS levels were normalized to the total protein content and expressed as micromoles of DCF per milligram of protein.

#### Total Sulfhydryl (SH) Content

The SH content in cell lysates was determined using the 5,5′-dithiobis­(2-nitrobenzoic acid) (DTNB) assay, as previously described by Aksenov and Markesbery,^75^ with minor modifications. This colorimetric method is based on the reaction between free thiol groups and DTNB, resulting in the formation of the yellow chromophore 5-thio-2-nitrobenzoic acid (TNB), which absorbs light at 412 nm. Briefly, cell lysates were diluted in phosphate-buffered saline (PBS, pH 7.4) containing 1 mM EDTA. The reaction was initiated by adding DTNB, and after sufficient incubation, the absorbance was recorded at 412 nm using a spectrophotometer (SpectraMax190). The concentration of SH groups was calculated using a standard curve of TNB and expressed as nanomoles of TNB per milligram of protein.

#### Superoxide Dismutase (SOD) Activity

The activity of SOD was assessed based on the method established by Misra and Fridovich,^76^ which involves the inhibition of adrenaline auto-oxidation by superoxide radicals. In this assay, cell lysates were first treated with catalase to eliminate potential interference from hydrogen peroxide (H_2_O_2_). Subsequently, glycine buffer (pH 10.2) and adrenaline were added to initiate the reaction. The formation of adrenochrome, resulting from the oxidation of adrenaline, was monitored by measuring the absorbance at 480 nm using a microplate reader (SpectraMax 190). The enzyme activity was calculated based on the inhibition rate of adrenaline oxidation and expressed as units per milligram of protein.

#### Catalase (CAT) Activity

CAT activity was determined following the protocol described by Aebi,^77^ which is based on the decomposition of H_2_O_2_ in a potassium phosphate buffer (pH 7.0). Briefly, the reaction was initiated by adding H_2_O_2_ to the cell lysates prepared in phosphate buffer. The breakdown of H_2_O_2_ was monitored by measuring the decrease in absorbance at 240 nm at 37 °C using a microplate reader (SpectraMax 190). Enzyme activity was calculated from the rate of H_2_O_2_ decomposition and expressed as units per milligram of protein.

#### Glutathione S-Transferase (GST) Activity

GST activity was determined using 1-chloro-2,4-dinitrobenzene (CDNB) as the substrate, following a previously established protocol.^78^ The reaction mixture consisted of 1 mM CDNB (dissolved in ethanol), 10 mM reduced glutathione (GSH), 20 mM potassium phosphate buffer (pH 6.5), and 20 μL of the sample. The formation of the GS-DNB conjugate was monitored spectrophotometrically (SpectraMax 190). Enzyme activity was expressed as micromoles of GS-DNB formed per minute per milligram of protein (μmol/min/mg protein).

#### Histopathological Evaluation

The posterior hind paws were collected post-mortem and fixed by immersion in 10% neutral-buffered formalin for 24 h. Following fixation, samples were decalcified in an 8% solution composed of equal parts hydrochloric acid and formic acid (1:1 ratio) for approximately 5 days. Decalcified tissues were then transversely sectioned, routinely processed, embedded in paraffin, and cut into sections 3–4 μm thick. Histological slides were stained with hematoxylin and eosin (H&E) and examined under light microscopy by two independent and blinded pathologists.

Skeletal muscle lesions were semiquantitatively scored based on the extent of myofiber necrosis and accompanying inflammatory infiltration. The grading criteria were defined as follows:

- Mild (+): up to 33% of muscle fibers showing necrosis, with multifocal inflammatory cell infiltrates interspersed among preserved fibers;
- Moderate (++): 34–66% of muscle fibers affected, with multifocal to coalescing inflammatory infiltrates;
- Severe (+++): more than 66% of muscle fibers exhibiting necrosis, with diffuse and widespread inflammatory infiltration throughout the muscle tissue.

This grading system enabled a consistent and reproducible assessment of tumor-associated muscle damage across experimental groups.

### Biochemical Assays

#### Tissue Preparation

On the day of euthanasia, samples from the cerebral cortex, spinal cord, cerebellum, and hippocampus were dissected. The collected tissues were immediately frozen and stored at −80 °C in an ultralow-temperature freezer. For the biochemical assays, samples were homogenized in 50 mM Tris–HCl buffer (pH 7.4) and subsequently centrifuged at 900*g* for 10 min to obtain the supernatant (S1). Protein concentration was determined spectrophotometrically at 595 nm using the Bradford assay,^79^ with bovine serum albumin (BSA) serving as the standard. Protein concentrations were expressed as mg/mL. These values were used for normalization in subsequent biochemical analyses.

#### TBARS Levels

TBARS levels were used as an indirect method to quantify the presence of malondialdehyde (MDA), one of the products formed during lipid peroxidation. The measurement of TBARS levels followed the protocol described by Ohkawa et al.^80^ An aliquot of S1 was added to a reaction mixture containing thiobarbituric acid (0.8%), sodium dodecyl sulfate (8.1%), and acetic acid (pH 3.4), and then incubated at 95 °C for 2 h. Subsequently, the absorbance was measured at 532 nm using a spectrophotometer. The results were reported as nmol MDA/mg protein.

#### Na^+^ K^+^-ATPase and Mg^2+^-ATPase Activities

The reaction mixture consisted of S1, 3 mM MgCl_2_, 125 mM NaCl, 20 mM KCl, and 50 mM Tris/HCl (pH 7.4), with a final volume of 500 μL. The reaction was initiated by adding ATP to a final concentration of 3.0 mM. Control samples were prepared under the same conditions with the addition of 0.1 mM ouabain. Ouabain is an inhibitor of the Na^+^, K^+^ pump, and its inclusion allowed the observation of enzyme activity related to the Mg^2+^ pump. To determine the Mg^2+^-ATPase activity, 1 mM ouabain was added to the reaction medium. The reactions were initiated by adding ATP and stopped after 30 min of incubation by the addition of 10% TCA.

The samples were further incubated at 37 °C for 30 min and the incubation was halted by adding 10% TCA with 10 mM HgCl_2_. Enzyme activity was calculated by measuring the difference in inorganic phosphate (Pi) levels between incubations conducted in the absence and presence of ouabain. The quantification of released inorganic phosphate (Pi) was performed using the method described by Fiske and Subbarow.^81^ The color reaction was spectrophotometrically analyzed at 650 nm. Results were expressed as nmol Pi/mg protein/min.

#### Statistical Analysis

Statistical analysis was performed using one-way ANOVA followed by Tukey’s posthoc test when appropriate. Data were presented as mean ± standard error of the mean (S.E.M.). Differences were considered significant at *P* < 0.05, using GraphPad Prism 9.5.0 and 8.0.1 software (GraphPad Prism, San Diego, USA). Post hoc comparisons were performed using Tukey’s multiple comparisons test, which is suitable for pairwise comparisons between group means while controlling the family wise error rate. This method was selected to appropriately address multiple comparisons and reduce the likelihood of type I errors.

Exact *p*-values were reported whenever possible, and differences were considered statistically significant when *p* < 0.05. Detailed results of the multiple comparisons, including mean differences, 95% confidence intervals, and adjusted *p*-values, are provided in the Supporting Information.

## Supplementary Material



## Notes

Detailed statistical results for tumor progression, nociceptive behavior, cytotoxicity, oxidative stress in macrophages, and biochemical assays; Tukey’s multiple comparisons test tables; general procedure for the synthesis of 7-chloro-4-(phenylselanyl)­quinoline; HRMS spectrum for 7-chloro-4-(phenylselanyl)­quinoline (PDF)
