# Supplementary material for: Therapeutic Efficacy of 7‑Chloro-4-(phenylselanyl)quinoline against Oncological Pain Induced by Tumor Progression and Vincristine Treatment
Source: ACS Omega. 2026 Jun 12;11(25):37298–315. doi: 10.1021/acsomega.5c11481 (PMC13325082; doi:10.1021/acsomega.5c11481)
Supplement: Supplementary file 1 [file ao5c11481_si_001.pdf]

Supplementary Material

Therapeutic Efficacy of 7-chloro-4-(phenylselanyl)quinoline Against Oncological Pain Induced by Tumor Progression and Vincristine Treatment

Ketlyn P. da Motta[a], Vanessa M. E. da Rocha[a], Bruna S. Pacheco[b], Tiago V. Collares [b], Fabiana K. Seixas[b], Joanna V.Z. Echenique[c], Mauro P. Soares[c], Rhayane Tavares[d], Diego Alves[d], Nathalia S. Pedra[e], Natália P. Bona[e], Roselia M. Spanevello[e], Juliano S. Barin[f], Pricila N. Pinheiro[ag], Vinicius C. Prado[a], Marcia F. Mesko[g] and Ethel A. Wilhelm [a*]

Detailed statistical results

Assessment of Tumor Progression

Width of the left hind paw

Table S1. Tukey's Multiple Comparisons Test for Group Differences Obtained Using GraphPad Prism 8.0

Tukey's multiple comparisons test	Mean Diff,	95,00% CI of diff,	Significant?	Summary	Adjusted P Value
		**Day 1**			
CONTROL vs. S180	-0,02500	-0,08871 to 0,03871	No	ns	0,7907
CONTROL vs. S180 + VCR	-0,04375	-0,1075 to 0,01996	No	ns	0,2993
CONTROL vs. S180 + 4-PSQ	-0,03750	-0,1012 to 0,02621	No	ns	0,4516
CONTROL vs. S180 + VCR + 4-PSQ	-0,02500	-0,08871 to 0,03871	No	ns	0,7907
S180 vs. S180 + VCR	-0,01875	-0,08246 to 0,04496	No	ns	0,9142
S180 vs. S180 + 4-PSQ	-0,01250	-0,07621 to 0,05121	No	ns	0,9794
S180 vs. S180 + VCR + 4-PSQ	0,000	-0,06371 to 0,06371	No	ns	>0,9999
S180 + VCR vs. S180 + 4-PSQ	0,006250	-0,05746 to 0,06996	No	ns	0,9985
S180 + VCR vs. S180 + VCR + 4-PSQ	0,01875	-0,04496 to 0,08246	No	ns	0,9142
S180 + 4-PSQ vs. S180 + VCR + 4-PSQ	0,01250	-0,05121 to 0,07621	No	ns	0,9794
		**Day 3**			
CONTROL vs. S180	-0,1143	-0,1667 to -0,06187	Yes	****	<0,0001
CONTROL vs. S180 + VCR	-0,1071	-0,1596 to -0,05473	Yes	****	<0,0001
CONTROL vs. S180 + 4-PSQ	-0,1000	-0,1524 to -0,04759	Yes	****	<0,0001
CONTROL vs. S180 + VCR + 4-PSQ	-0,08571	-0,1381 to -0,03330	Yes	***	0,0004
S180 vs. S180 + VCR	0,007143	-0,04527 to 0,05956	No	ns	0,9946
S180 vs. S180 + 4-PSQ	0,01429	-0,03813 to 0,06670	No	ns	0,9314
S180 vs. S180 + VCR + 4-PSQ	0,02857	-0,02384 to 0,08099	No	ns	0,5204
S180 + VCR vs. S180 + 4-PSQ	0,007143	-0,04527 to 0,05956	No	ns	0,9946
S180 + VCR vs. S180 + VCR + 4-PSQ	0,02143	-0,03099 to 0,07384	No	ns	0,7592
S180 + 4-PSQ vs. S180 + VCR + 4-PSQ	0,01429	-0,03813 to 0,06670	No	ns	0,9314
		**Day 5**			
CONTROL vs. S180	-0,09286	-0,1442 to -0,04155	Yes	***	0,0001
CONTROL vs. S180 + VCR	-0,1143	-0,1656 to -0,06297	Yes	****	<0,0001
CONTROL vs. S180 + 4-PSQ	-0,07143	-0,1227 to -0,02012	Yes	**	0,0029

CONTROL vs. S180 + VCR + 4-PSQ	-0,07857	-0,1299 to -0,02726	Yes	***	0,0010
S180 vs. S180 + VCR	-0,02143	-0,07274 to 0,02988	No	ns	0,7449
S180 vs. S180 + 4-PSQ	0,02143	-0,02988 to 0,07274	No	ns	0,7449
S180 vs. S180 + VCR + 4-PSQ	0,01429	-0,03703 to 0,06560	No	ns	0,9262
S180 + VCR vs. S180 + 4-PSQ	0,04286	-0,008454 to 0,09417	No	ns	0,1369
S180 + VCR vs. S180 + VCR + 4-PSQ	0,03571	-0,01560 to 0,08703	No	ns	0,2817
S180 + 4-PSQ vs. S180 + VCR + 4-PSQ	-0,007143	-0,05845 to 0,04417	No	ns	0,9941
**Day 7**					
CONTROL vs. S180	-0,1929	-0,2329 to -0,1528	Yes	****	<0,0001
CONTROL vs. S180 + VCR	-0,1000	-0,1400 to -0,05997	Yes	****	<0,0001
CONTROL vs. S180 + 4-PSQ	-0,1786	-0,2186 to -0,1385	Yes	****	<0,0001
CONTROL vs. S180 + VCR + 4-PSQ	-0,1143	-0,1543 to -0,07425	Yes	****	<0,0001
S180 vs. S180 + VCR	0,09286	0,05282 to 0,1329	Yes	****	<0,0001
S180 vs. S180 + 4-PSQ	0,01429	-0,02575 to 0,05432	No	ns	0,8371
S180 vs. S180 + VCR + 4-PSQ	0,07857	0,03854 to 0,1186	Yes	****	<0,0001
S180 + VCR vs. S180 + 4-PSQ	-0,07857	-0,1186 to -0,03854	Yes	****	<0,0001
S180 + VCR vs. S180 + VCR + 4-PSQ	-0,01429	-0,05432 to 0,02575	No	ns	0,8371
S180 + 4-PSQ vs. S180 + VCR + 4-PSQ	0,06429	0,02425 to 0,1043	Yes	***	0,0005
**Day 9**					
CONTROL vs. S180	-0,1714	-0,2432 to -0,09966	Yes	****	<0,0001
CONTROL vs. S180 + VCR	-0,1143	-0,1861 to -0,04251	Yes	***	0,0006
CONTROL vs. S180 + 4-PSQ	-0,1571	-0,2289 to -0,08537	Yes	****	<0,0001
CONTROL vs. S180 + VCR + 4-PSQ	-0,1429	-0,2146 to -0,07109	Yes	****	<0,0001
S180 vs. S180 + VCR	0,05714	-0,01463 to 0,1289	No	ns	0,1699
S180 vs. S180 + 4-PSQ	0,01429	-0,05749 to 0,08606	No	ns	0,9774
S180 vs. S180 + VCR + 4-PSQ	0,02857	-0,04320 to 0,1003	No	ns	0,7763
S180 + VCR vs. S180 + 4-PSQ	-0,04286	-0,1146 to 0,02891	No	ns	0,4305
S180 + VCR vs. S180 + VCR + 4-PSQ	-0,02857	-0,1003 to 0,04320	No	ns	0,7763
S180 + 4-PSQ vs. S180 + VCR + 4-PSQ	0,01429	-0,05749 to 0,08606	No	ns	0,9774
**Day 11**					
CONTROL vs. S180	-0,2357	-0,3008 to -0,1706	Yes	****	<0,0001
CONTROL vs. S180 + VCR	-0,1429	-0,2079 to -0,07778	Yes	****	<0,0001
CONTROL vs. S180 + 4-PSQ	-0,1643	-0,2294 to -0,09921	Yes	****	<0,0001
CONTROL vs. S180 + VCR + 4-PSQ	-0,1143	-0,1794 to -0,04921	Yes	***	0,0002
S180 vs. S180 + VCR	0,09286	0,02778 to 0,1579	Yes	**	0,0023
S180 vs. S180 + 4-PSQ	0,07143	0,006349 to 0,1365	Yes	*	0,0259
S180 vs. S180 + VCR + 4-PSQ	0,1214	0,05635 to 0,1865	Yes	****	<0,0001
S180 + VCR vs. S180 + 4-PSQ	-0,02143	-0,08651 to 0,04365	No	ns	0,8727
S180 + VCR vs. S180 + VCR + 4-PSQ	0,02857	-0,03651 to 0,09365	No	ns	0,7090
S180 + 4-PSQ vs. S180 + VCR + 4-PSQ	0,05000	-0,01508 to 0,1151	No	ns	0,1970
**Day 13**					
CONTROL vs. S180	-0,2943	-0,3773 to -0,2112	Yes	****	<0,0001
CONTROL vs. S180 + VCR	-0,2500	-0,3331 to -0,1669	Yes	****	<0,0001
CONTROL vs. S180 + 4-PSQ	-0,2143	-0,2973 to -0,1312	Yes	****	<0,0001
CONTROL vs. S180 + VCR + 4-PSQ	-0,1429	-0,2259 to -0,05980	Yes	***	0,0002
S180 vs. S180 + VCR	0,04429	-0,03877 to 0,1273	No	ns	0,5416
S180 vs. S180 + 4-PSQ	0,08000	-0,003054 to 0,1631	No	ns	0,0634
S180 vs. S180 + VCR + 4-PSQ	0,1514	0,06837 to 0,2345	Yes	****	<0,0001
S180 + VCR vs. S180 + 4-PSQ	0,03571	-0,04734 to 0,1188	No	ns	0,7243
S180 + VCR vs. S180 + VCR + 4-PSQ	0,1071	0,02409 to 0,1902	Yes	**	0,0064
S180 + 4-PSQ vs. S180 + VCR + 4-PSQ	0,07143	-0,01163 to 0,1545	No	ns	0,1188
**Day 15**					
CONTROL vs. S180	-0,2657	-0,3547 to -0,1767	Yes	****	<0,0001
CONTROL vs. S180 + VCR	-0,2214	-0,3104 to -0,1325	Yes	****	<0,0001
CONTROL vs. S180 + 4-PSQ	-0,1714	-0,2604 to -0,08245	Yes	****	<0,0001
CONTROL vs. S180 + VCR + 4-PSQ	-0,1000	-0,1890 to -0,01102	Yes	*	0,0216

S180 vs. S180 + VCR	0,04429	-0,04469 to 0,1333	No	ns	0,6054
S180 vs. S180 + 4-PSQ	0,09429	0,005310 to 0,1833	Yes	*	0,0336
S180 vs. S180 + VCR + 4-PSQ	0,1657	0,07674 to 0,2547	Yes	****	<0,0001
S180 + VCR vs. S180 + 4-PSQ	0,05000	-0,03898 to 0,1390	No	ns	0,4907
S180 + VCR vs. S180 + VCR + 4-PSQ	0,1214	0,03245 to 0,2104	Yes	**	0,0036
S180 + 4-PSQ vs. S180 + VCR + 4-PSQ	0,07143	-0,01755 to 0,1604	No	ns	0,1639
**Day 17**					
CONTROL vs. S180	-0,2071	-0,2793 to -0,1350	Yes	****	<0,0001
CONTROL vs. S180 + VCR	-0,2000	-0,2722 to -0,1278	Yes	****	<0,0001
CONTROL vs. S180 + 4-PSQ	-0,1643	-0,2365 to -0,09212	Yes	****	<0,0001
CONTROL vs. S180 + VCR + 4-PSQ	-0,1071	-0,1793 to -0,03497	Yes	**	0,0014
S180 vs. S180 + VCR	0,007143	-0,06503 to 0,07931	No	ns	0,9984
S180 vs. S180 + 4-PSQ	0,04286	-0,02931 to 0,1150	No	ns	0,4360
S180 vs. S180 + VCR + 4-PSQ	0,1000	0,02783 to 0,1722	Yes	**	0,0031
S180 + VCR vs. S180 + 4-PSQ	0,03571	-0,03645 to 0,1079	No	ns	0,6105
S180 + VCR vs. S180 + VCR + 4-PSQ	0,09286	0,02069 to 0,1650	Yes	**	0,0066
S180 + 4-PSQ vs. S180 + VCR + 4-PSQ	0,05714	-0,01503 to 0,1293	No	ns	0,1739

Data are presented as results of Tukey's multiple comparisons post hoc test following one-way ANOVA, performed using GraphPad Prism version 8.0.

Abbreviations: Mean Diff, mean difference between groups; 95% CI of diff, 95% confidence interval of the difference; Significant, statistical significance of the comparison; Summary, significance notation; Adjusted P Value, p-value corrected for multiple comparisons.

Statistical significance is indicated as follows: *$p < 0.05$, **$p < 0.01$, ***$p < 0.001$, and ****$p < 0.0001$.

Depth of the left hind paw

Table S2. Tukey's Multiple Comparisons Test for Group Differences Obtained Using GraphPad Prism 8.0

Tukey's multiple comparisons test	Mean Diff,	95,00% CI of diff,	Significant?	Summary	Adjusted P Value
**Day 1**					
CONTROL vs. S180	-0,05000	-0,1293 to 0,02935	No	ns	0,3771
CONTROL vs. S180 + VCR	-0,04286	-0,1222 to 0,03649	No	ns	0,5293
CONTROL vs. S180 + 4-PSQ	-0,05714	-0,1365 to 0,02220	No	ns	0,2511
CONTROL vs. S180 + VCR + 4-PSQ	-0,07857	-0,1579 to 0,0007749	No	ns	0,0533
S180 vs. S180 + VCR	0,007143	-0,07220 to 0,08649	No	ns	0,9989
S180 vs. S180 + 4-PSQ	-0,007143	-0,08649 to 0,07220	No	ns	0,9989
S180 vs. S180 + VCR + 4-PSQ	-0,02857	-0,1079 to 0,05077	No	ns	0,8326
S180 + VCR vs. S180 + 4-PSQ	-0,01429	-0,09363 to 0,06506	No	ns	0,9844
S180 + VCR vs. S180 + VCR + 4-PSQ	-0,03571	-0,1151 to 0,04363	No	ns	0,6899
S180 + 4-PSQ vs. S180 + VCR + 4-PSQ	-0,02143	-0,1008 to 0,05792	No	ns	0,9335
**Day 3**					
CONTROL vs. S180	-0,04286	-0,08540 to -0,0003109	Yes	*	0,0477
CONTROL vs. S180 + VCR	0,007143	-0,03680 to 0,05108	No	ns	0,9895
CONTROL vs. S180 + 4-PSQ	-0,01429	-0,05823 to 0,02966	No	ns	0,8786
CONTROL vs. S180 + VCR + 4-PSQ	-0,007143	-0,05108 to 0,03680	No	ns	0,9895
S180 vs. S180 + VCR	0,05000	0,007454 to 0,09255	Yes	*	0,0149
S180 vs. S180 + 4-PSQ	0,02857	-0,01397 to 0,07112	No	ns	0,3165
S180 vs. S180 + VCR + 4-PSQ	0,03571	-0,006832 to 0,07826	No	ns	0,1341
S180 + VCR vs. S180 + 4-PSQ	-0,02143	-0,06537 to 0,02251	No	ns	0,6250
S180 + VCR vs. S180 + VCR + 4-PSQ	-0,01429	-0,05823 to 0,02966	No	ns	0,8786
S180 + 4-PSQ vs. S180 + VCR + 4-PSQ	0,007143	-0,03680 to 0,05108	No	ns	0,9895
**Day 5**					

CONTROL vs. S180	-0,07857	-0,1495 to -0,007602	Yes	*	0,0242
CONTROL vs. S180 + VCR	-0,07143	-0,1424 to -0,0004591	Yes	*	0,0479
CONTROL vs. S180 + 4-PSQ	-0,07857	-0,1495 to -0,007602	Yes	*	0,0242
CONTROL vs. S180 + VCR + 4-PSQ	-0,07143	-0,1424 to -0,0004591	Yes	*	0,0479
S180 vs. S180 + VCR	0,007143	-0,06383 to 0,07811	No	ns	0,9983
S180 vs. S180 + 4-PSQ	0,000	-0,07097 to 0,07097	No	ns	>0,9999
S180 vs. S180 + VCR + 4-PSQ	0,007143	-0,06383 to 0,07811	No	ns	0,9983
S180 + VCR vs. S180 + 4-PSQ	-0,007143	-0,07811 to 0,06383	No	ns	0,9983
S180 + VCR vs. S180 + VCR + 4-PSQ	0,000	-0,07097 to 0,07097	No	ns	>0,9999
S180 + 4-PSQ vs. S180 + VCR + 4-PSQ	0,007143	-0,06383 to 0,07811	No	ns	0,9983
**Day 7**					
CONTROL vs. S180	-0,2071	-0,2761 to -0,1382	Yes	****	<0,0001
CONTROL vs. S180 + VCR	-0,1071	-0,1761 to -0,03822	Yes	***	0,0008
CONTROL vs. S180 + 4-PSQ	-0,1571	-0,2261 to -0,08822	Yes	****	<0,0001
CONTROL vs. S180 + VCR + 4-PSQ	-0,1500	-0,2189 to -0,08108	Yes	****	<0,0001
S180 vs. S180 + VCR	0,1000	0,03108 to 0,1689	Yes	**	0,0019
S180 vs. S180 + 4-PSQ	0,05000	-0,01892 to 0,1189	No	ns	0,2447
S180 vs. S180 + VCR + 4-PSQ	0,05714	-0,01178 to 0,1261	No	ns	0,1417
S180 + VCR vs. S180 + 4-PSQ	-0,05000	-0,1189 to 0,01892	No	ns	0,2447
S180 + VCR vs. S180 + VCR + 4-PSQ	-0,04286	-0,1118 to 0,02607	No	ns	0,3903
S180 + 4-PSQ vs. S180 + VCR + 4-PSQ	0,007143	-0,06178 to 0,07607	No	ns	0,9981
**Day 9**					
CONTROL vs. S180	-0,2286	-0,3178 to -0,1394	Yes	****	<0,0001
CONTROL vs. S180 + VCR	-0,07857	-0,1678 to 0,01062	No	ns	0,1051
CONTROL vs. S180 + 4-PSQ	-0,1786	-0,2678 to -0,08938	Yes	****	<0,0001
CONTROL vs. S180 + VCR + 4-PSQ	-0,1857	-0,2749 to -0,09652	Yes	****	<0,0001
S180 vs. S180 + VCR	0,1500	0,06081 to 0,2392	Yes	***	0,0003
S180 vs. S180 + 4-PSQ	0,05000	-0,03919 to 0,1392	No	ns	0,4931
S180 vs. S180 + VCR + 4-PSQ	0,04286	-0,04634 to 0,1321	No	ns	0,6363
S180 + VCR vs. S180 + 4-PSQ	-0,1000	-0,1892 to -0,01081	Yes	*	0,0220
S180 + VCR vs. S180 + VCR + 4-PSQ	-0,1071	-0,1963 to -0,01795	Yes	*	0,0124
S180 + 4-PSQ vs. S180 + VCR + 4-PSQ	-0,007143	-0,09634 to 0,08205	No	ns	0,9993
**Day 11**					
CONTROL vs. S180	-0,2286	-0,3298 to -0,1274	Yes	****	<0,0001
CONTROL vs. S180 + VCR	-0,1786	-0,2798 to -0,07735	Yes	***	0,0002
CONTROL vs. S180 + 4-PSQ	-0,2286	-0,3298 to -0,1274	Yes	****	<0,0001
CONTROL vs. S180 + VCR + 4-PSQ	-0,2000	-0,3012 to -0,09878	Yes	****	<0,0001
S180 vs. S180 + VCR	0,05000	-0,05122 to 0,1512	No	ns	0,6121
S180 vs. S180 + 4-PSQ	0,000	-0,1012 to 0,1012	No	ns	>0,9999
S180 vs. S180 + VCR + 4-PSQ	0,02857	-0,07265 to 0,1298	No	ns	0,9228
S180 + VCR vs. S180 + 4-PSQ	-0,05000	-0,1512 to 0,05122	No	ns	0,6121
S180 + VCR vs. S180 + VCR + 4-PSQ	-0,02143	-0,1226 to 0,07979	No	ns	0,9717
S180 + 4-PSQ vs. S180 + VCR + 4-PSQ	0,02857	-0,07265 to 0,1298	No	ns	0,9228
**Day 13**					
CONTROL vs. S180	-0,3086	-0,4326 to -0,1846	Yes	****	<0,0001
CONTROL vs. S180 + VCR	-0,1929	-0,3169 to -0,06884	Yes	***	0,0008
CONTROL vs. S180 + 4-PSQ	-0,1786	-0,3026 to -0,05455	Yes	**	0,0020
CONTROL vs. S180 + VCR + 4-PSQ	-0,1571	-0,2812 to -0,03313	Yes	**	0,0076
S180 vs. S180 + VCR	0,1157	-0,008302 to 0,2397	No	ns	0,0766
S180 vs. S180 + 4-PSQ	0,1300	0,005983 to 0,2540	Yes	*	0,0363
S180 vs. S180 + VCR + 4-PSQ	0,1514	0,02741 to 0,2754	Yes	*	0,0107
S180 + VCR vs. S180 + 4-PSQ	0,01429	-0,1097 to 0,1383	No	ns	0,9972
S180 + VCR vs. S180 + VCR + 4-PSQ	0,03571	-0,08830 to 0,1597	No	ns	0,9174
S180 + 4-PSQ vs. S180 + VCR + 4-PSQ	0,02143	-0,1026 to 0,1454	No	ns	0,9866
**Day 15**					
CONTROL vs. S180	-0,3229	-0,4417 to -0,2040	Yes	****	<0,0001

CONTROL vs. S180 + VCR	-0,2429	-0,3617 to -0,1240	Yes	****	<0,0001
CONTROL vs. S180 + 4-PSQ	-0,2143	-0,3331 to -0,09545	Yes	***	0,0001
CONTROL vs. S180 + VCR + 4-PSQ	-0,1429	-0,2617 to -0,02403	Yes	*	0,0123
S180 vs. S180 + VCR	0,08000	-0,03883 to 0,1988	No	ns	0,3129
S180 vs. S180 + 4-PSQ	0,1086	-0,01026 to 0,2274	No	ns	0,0863
S180 vs. S180 + VCR + 4-PSQ	0,1800	0,06117 to 0,2988	Yes	**	0,0011
S180 + VCR vs. S180 + 4-PSQ	0,02857	-0,09026 to 0,1474	No	ns	0,9555
S180 + VCR vs. S180 + VCR + 4-PSQ	0,1000	-0,01883 to 0,2188	No	ns	0,1321
S180 + 4-PSQ vs. S180 + VCR + 4-PSQ	0,07143	-0,04740 to 0,1903	No	ns	0,4240
**Day 17**					
CONTROL vs. S180	-0,2943	-0,4580 to -0,1305	Yes	***	0,0001
CONTROL vs. S180 + VCR	-0,2214	-0,3852 to -0,05767	Yes	**	0,0040
CONTROL vs. S180 + 4-PSQ	-0,1786	-0,3423 to -0,01482	Yes	*	0,0272
CONTROL vs. S180 + VCR + 4-PSQ	-0,1286	-0,2923 to 0,03518	No	ns	0,1802
S180 vs. S180 + VCR	0,07286	-0,09090 to 0,2366	No	ns	0,6989
S180 vs. S180 + 4-PSQ	0,1157	-0,04804 to 0,2795	No	ns	0,2680
S180 vs. S180 + VCR + 4-PSQ	0,1657	0,001959 to 0,3295	Yes	*	0,0462
S180 + VCR vs. S180 + 4-PSQ	0,04286	-0,1209 to 0,2066	No	ns	0,9402
S180 + VCR vs. S180 + VCR + 4-PSQ	0,09286	-0,07090 to 0,2566	No	ns	0,4818
S180 + 4-PSQ vs. S180 + VCR + 4-PSQ	0,05000	-0,1138 to 0,2138	No	ns	0,9000

Data are presented as results of Tukey’s multiple comparisons post hoc test following one-way ANOVA, performed using GraphPad Prism version 8.0.

Abbreviations: Mean Diff, mean difference between groups; 95% CI of diff, 95% confidence interval of the difference; Significant, statistical significance of the comparison; Summary, significance notation; Adjusted P Value, p-value corrected for multiple comparisons.

Statistical significance is indicated as follows: *p < 0.05, **p < 0.01, ***p < 0.001, and ****p < 0.0001.

Paw edema

Table S3. Tukey’s Multiple Comparisons Test for Group Differences Obtained Using GraphPad Prism 8.0

Tukey's multiple comparisons test	Mean Diff,	95,00% CI of diff,	Significant?	Summary
CONTROL vs. S180	-0,1772	-0,3044 to -0,05005	Yes	**
CONTROL vs. S180 + VCR	0,003386	-0,1238 to 0,1305	No	ns
CONTROL vs. S180 + 4-PSQ	-0,02633	-0,1535 to 0,1008	No	ns
CONTROL vs. S180 + VCR + 4-PSQ	-0,008486	-0,1356 to 0,1187	No	ns
S180 vs. S180 + VCR	0,1806	0,05343 to 0,3078	Yes	**
S180 vs. S180 + 4-PSQ	0,1509	0,02372 to 0,2780	Yes	*
S180 vs. S180 + VCR + 4-PSQ	0,1687	0,04156 to 0,2959	Yes	**
S180 + VCR vs. S180 + 4-PSQ	-0,02971	-0,1569 to 0,09745	No	ns
S180 + VCR vs. S180 + VCR + 4-PSQ	-0,01187	-0,1390 to 0,1153	No	ns
S180 + 4-PSQ vs. S180 + VCR + 4-PSQ	0,01784	-0,1093 to 0,1450	No	ns

Data are presented as results of Tukey’s multiple comparisons post hoc test following one-way ANOVA, performed using GraphPad Prism version 8.0.

Abbreviations: Mean Diff, mean difference between groups; 95% CI of diff, 95% confidence interval of the difference; Significant, statistical significance of the comparison; Summary, significance notation; Adjusted P Value, p-value corrected for multiple comparisons.

Statistical significance is indicated as follows: *p < 0.05 and **p < 0.01.

Nociception evaluation

Assessment of Mechanical Nociceptive Threshold

Table S4. Tukey's Multiple Comparisons Test for Group Differences Obtained Using GraphPad Prism 8.0

Tukey's multiple comparisons test	Mean Diff,	95,00% CI of diff,	Significant?	Summary	Adjusted P Value
		**Basal**			
CONTROL vs. S180	-0,08125	-0,5834 to 0,4209	No	ns	0,9900
CONTROL vs. S180 + VCR	-0,1125	-0,6146 to 0,3896	No	ns	0,9666
CONTROL vs. S180 + 4-PSQ	0,2463	-0,2559 to 0,7484	No	ns	0,6255
CONTROL vs. S180 + VCR + 4-PSQ	0,1300	-0,3721 to 0,6321	No	ns	0,9444
S180 vs. S180 + VCR	-0,03125	-0,5334 to 0,4709	No	ns	0,9998
S180 vs. S180 + 4-PSQ	0,3275	-0,1746 to 0,8296	No	ns	0,3491
S180 vs. S180 + VCR + 4-PSQ	0,2113	-0,2909 to 0,7134	No	ns	0,7459
S180 + VCR vs. S180 + 4-PSQ	0,3588	-0,1434 to 0,8609	No	ns	0,2625
S180 + VCR vs. S180 + VCR + 4-PSQ	0,2425	-0,2596 to 0,7446	No	ns	0,6388
S180 + 4-PSQ vs. S180 + VCR + 4-PSQ	-0,1163	-0,6184 to 0,3859	No	ns	0,9624
		**Day 7**			
CONTROL vs. S180	5,913	5,272 to 6,553	Yes	****	<0,0001
CONTROL vs. S180 + VCR	7,710	7,070 to 8,350	Yes	****	<0,0001
CONTROL vs. S180 + 4-PSQ	5,238	4,597 to 5,878	Yes	****	<0,0001
CONTROL vs. S180 + VCR + 4-PSQ	7,545	6,905 to 8,185	Yes	****	<0,0001
S180 vs. S180 + VCR	1,798	1,157 to 2,438	Yes	****	<0,0001
S180 vs. S180 + 4-PSQ	-0,6750	-1,315 to -0,03454	Yes	*	0,0347
S180 vs. S180 + VCR + 4-PSQ	1,633	0,9920 to 2,273	Yes	****	<0,0001
S180 + VCR vs. S180 + 4-PSQ	-2,473	-3,113 to -1,832	Yes	****	<0,0001
S180 + VCR vs. S180 + VCR + 4-PSQ	-0,1650	-0,8055 to 0,4755	No	ns	0,9453
S180 + 4-PSQ vs. S180 + VCR + 4-PSQ	2,308	1,667 to 2,948	Yes	****	<0,0001
		**Day 11**			
CONTROL vs. S180	5,021	4,402 to 5,640	Yes	****	<0,0001
CONTROL vs. S180 + VCR	7,571	6,952 to 8,190	Yes	****	<0,0001
CONTROL vs. S180 + 4-PSQ	2,046	1,427 to 2,665	Yes	****	<0,0001
CONTROL vs. S180 + VCR + 4-PSQ	1,613	0,9937 to 2,231	Yes	****	<0,0001
S180 vs. S180 + VCR	2,550	1,931 to 3,169	Yes	****	<0,0001
S180 vs. S180 + 4-PSQ	-2,975	-3,594 to -2,356	Yes	****	<0,0001
S180 vs. S180 + VCR + 4-PSQ	-3,409	-4,028 to -2,790	Yes	****	<0,0001
S180 + VCR vs. S180 + 4-PSQ	-5,525	-6,144 to -4,906	Yes	****	<0,0001
S180 + VCR vs. S180 + VCR + 4-PSQ	-5,959	-6,578 to -5,340	Yes	****	<0,0001
S180 + 4-PSQ vs. S180 + VCR + 4-PSQ	-0,4337	-1,053 to 0,1851	No	ns	0,2801
		**Day 17**			
CONTROL vs. S180	5,400	4,841 to 5,959	Yes	****	<0,0001
CONTROL vs. S180 + VCR	8,240	7,681 to 8,799	Yes	****	<0,0001
CONTROL vs. S180 + 4-PSQ	2,244	1,685 to 2,803	Yes	****	<0,0001
CONTROL vs. S180 + VCR + 4-PSQ	1,550	0,9908 to 2,109	Yes	****	<0,0001
S180 vs. S180 + VCR	2,840	2,281 to 3,399	Yes	****	<0,0001
S180 vs. S180 + 4-PSQ	-3,156	-3,715 to -2,597	Yes	****	<0,0001
S180 vs. S180 + VCR + 4-PSQ	-3,850	-4,409 to -3,291	Yes	****	<0,0001
S180 + VCR vs. S180 + 4-PSQ	-5,996	-6,555 to -5,437	Yes	****	<0,0001
S180 + VCR vs. S180 + VCR + 4-PSQ	-6,690	-7,249 to -6,131	Yes	****	<0,0001
S180 + 4-PSQ vs. S180 + VCR + 4-PSQ	-0,6938	-1,253 to -0,1345	Yes	**	0,0089

Data are presented as results of Tukey's multiple comparisons post hoc test following one-way ANOVA, performed using GraphPad Prism version 8.0.

Abbreviations: Mean Diff, mean difference between groups; 95% CI of diff, 95% confidence interval of the difference; Significant, statistical significance of the comparison; Summary, significance notation; Adjusted P Value, p-value corrected for multiple comparisons.

Statistical significance is indicated as follows: *$p < 0.05$, **$p < 0.01$, ***$p < 0.001$, and ****$p < 0.0001$.

Assessment of Thermal Nociceptive Threshold

Table S5. Tukey's Multiple Comparisons Test for Group Differences Obtained Using GraphPad Prism 8.0

Tukey's multiple comparisons test	Mean Diff,	95,00% CI of diff,	Significant?	Summary	Adjusted P Value
		**Basal**			
CONTROL vs. S180	-0,5714	-7,613 to 6,470	No	ns	0,9993
CONTROL vs. S180 + VCR	2,429	-4,613 to 9,470	No	ns	0,8531
CONTROL vs. S180 + 4-PSQ	4,000	-3,042 to 11,04	No	ns	0,4801
CONTROL vs. S180 + VCR + 4-PSQ	0,7143	-6,328 to 7,756	No	ns	0,9983
S180 vs. S180 + VCR	3,000	-4,042 to 10,04	No	ns	0,7310
S180 vs. S180 + 4-PSQ	4,571	-2,470 to 11,61	No	ns	0,3479
S180 vs. S180 + VCR + 4-PSQ	1,286	-5,756 to 8,328	No	ns	0,9836
S180 + VCR vs. S180 + 4-PSQ	1,571	-5,470 to 8,613	No	ns	0,9658
S180 + VCR vs. S180 + VCR + 4-PSQ	-1,714	-8,756 to 5,328	No	ns	0,9535
S180 + 4-PSQ vs. S180 + VCR + 4-PSQ	-3,286	-10,33 to 3,756	No	ns	0,6610
		**Day 7**			
CONTROL vs. S180	12,00	4,516 to 19,48	Yes	***	0,0005
CONTROL vs. S180 + VCR	27,63	20,14 to 35,11	Yes	****	<0,0001
CONTROL vs. S180 + 4-PSQ	11,38	3,891 to 18,86	Yes	***	0,0009
CONTROL vs. S180 + VCR + 4-PSQ	20,88	13,39 to 28,36	Yes	****	<0,0001
S180 vs. S180 + VCR	15,63	8,141 to 23,11	Yes	****	<0,0001
S180 vs. S180 + 4-PSQ	-0,6250	-8,109 to 6,859	No	ns	0,9992
S180 vs. S180 + VCR + 4-PSQ	8,875	1,391 to 16,36	Yes	*	0,0134
S180 + VCR vs. S180 + 4-PSQ	-16,25	-23,73 to -8,766	Yes	****	<0,0001
S180 + VCR vs. S180 + VCR + 4-PSQ	-6,750	-14,23 to 0,7344	No	ns	0,0936
S180 + 4-PSQ vs. S180 + VCR + 4-PSQ	9,500	2,016 to 16,98	Yes	**	0,0071
		**Day 11**			
CONTROL vs. S180	19,88	9,591 to 30,16	Yes	****	<0,0001
CONTROL vs. S180 + VCR	19,25	8,966 to 29,53	Yes	****	<0,0001
CONTROL vs. S180 + 4-PSQ	7,125	-3,159 to 17,41	No	ns	0,2909
CONTROL vs. S180 + VCR + 4-PSQ	1,375	-8,909 to 11,66	No	ns	0,9952
S180 vs. S180 + VCR	-0,6250	-10,91 to 9,659	No	ns	0,9998
S180 vs. S180 + 4-PSQ	-12,75	-23,03 to -2,466	Yes	**	0,0089
S180 vs. S180 + VCR + 4-PSQ	-18,50	-28,78 to -8,216	Yes	****	<0,0001
S180 + VCR vs. S180 + 4-PSQ	-12,13	-22,41 to -1,841	Yes	*	0,0141
S180 + VCR vs. S180 + VCR + 4-PSQ	-17,88	-28,16 to -7,591	Yes	***	0,0001
S180 + 4-PSQ vs. S180 + VCR + 4-PSQ	-5,750	-16,03 to 4,534	No	ns	0,5027
		**Day 17**			
CONTROL vs. S180	10,50	-4,708 to 25,71	No	ns	0,2824
CONTROL vs. S180 + VCR	22,33	7,126 to 37,54	Yes	**	0,0019
CONTROL vs. S180 + 4-PSQ	16,67	1,459 to 31,87	Yes	*	0,0267
CONTROL vs. S180 + VCR + 4-PSQ	1,833	-13,37 to 17,04	No	ns	0,9964
S180 vs. S180 + VCR	11,83	-3,374 to 27,04	No	ns	0,1829
S180 vs. S180 + 4-PSQ	6,167	-9,041 to 21,37	No	ns	0,7564
S180 vs. S180 + VCR + 4-PSQ	-8,667	-23,87 to 6,541	No	ns	0,4673
S180 + VCR vs. S180 + 4-PSQ	-5,667	-20,87 to 9,541	No	ns	0,8077
S180 + VCR vs. S180 + VCR + 4-PSQ	-20,50	-35,71 to -5,292	Yes	**	0,0046

S180 + 4-PSQ vs. S180 + VCR + 4-PSQ	-14,83	-30,04 to 0,3743	No	ns	0,0584

Data are presented as results of Tukey's multiple comparisons post hoc test following one-way ANOVA, performed using GraphPad Prism version 8.0.

Abbreviations: Mean Diff, mean difference between groups; 95% CI of diff, 95% confidence interval of the difference; Significant, statistical significance of the comparison; Summary, significance notation; Adjusted P Value, p-value corrected for multiple comparisons.

Statistical significance is indicated as follows: *p < 0.05, **p < 0.01, ***p < 0.001, and ****p < 0.0001.

Cytotoxicity

Cell viability

Table S6. Tukey's Multiple Comparisons Test for Group Differences Obtained Using GraphPad Prism 8.0

Tukey's multiple comparisons test	Mean Diff,	95,00% CI of diff,	Significant?	Summary	Adjusted P Value
CONTROL vs. S180	-28,62	-55,92 to -1,313	Yes	*	0,0371
CONTROL vs. VCR	10,59	-16,71 to 37,90	No	ns	0,7726
CONTROL vs. 4-PSQ	16,36	-10,94 to 43,66	No	ns	0,4047
CONTROL vs. VCR + 4-PSQ	9,964	-17,34 to 37,27	No	ns	0,8085
S180 vs. VCR	39,21	11,91 to 66,51	Yes	**	0,0029
S180 vs. 4-PSQ	44,97	17,67 to 72,28	Yes	***	0,0007
S180 vs. VCR + 4-PSQ	38,58	11,28 to 65,88	Yes	**	0,0034
VCR vs. 4-PSQ	5,764	-21,54 to 33,07	No	ns	0,9681
VCR vs. VCR + 4-PSQ	-0,6301	-27,93 to 26,67	No	ns	>0,9999
4-PSQ vs. VCR + 4-PSQ	-6,394	-33,70 to 20,91	No	ns	0,9539

Data are presented as results of Tukey's multiple comparisons post hoc test following one-way ANOVA, performed using GraphPad Prism version 8.0.

Abbreviations: Mean Diff, mean difference between groups; 95% CI of diff, 95% confidence interval of the difference; Significant, statistical significance of the comparison; Summary, significance notation; Adjusted P Value, p-value corrected for multiple comparisons.

Statistical significance is indicated as follows: *p < 0.05, **p < 0.01 and ***p < 0.001.

Cell Proliferation

Table S7. Tukey's Multiple Comparisons Test for Group Differences Obtained Using GraphPad Prism 8.0

Tukey's multiple comparisons test	Mean Diff,	95,00% CI of diff,	Significant?	Summary	Adjusted P Value
CONTROL vs. S180	81,90	21,08 to 142,7	Yes	**	0,0053
CONTROL vs. VCR	3,396	-57,42 to 64,22	No	ns	0,9998
CONTROL vs. 4-PSQ	-29,97	-90,79 to 30,85	No	ns	0,5896
CONTROL vs. VCR + 4-PSQ	31,12	-29,70 to 91,94	No	ns	0,5554
S180 vs. VCR	-78,50	-139,3 to -17,68	Yes	**	0,0077
S180 vs. 4-PSQ	-111,9	-172,7 to -51,05	Yes	***	0,0002
S180 vs. VCR + 4-PSQ	-50,78	-111,6 to 10,04	No	ns	0,1308
VCR vs. 4-PSQ	-33,36	-94,18 to 27,46	No	ns	0,4898
VCR vs. VCR + 4-PSQ	27,72	-33,10 to 88,54	No	ns	0,6563
4-PSQ vs. VCR + 4-PSQ	61,09	0,2677 to 121,9	Yes	*	0,0487

Data are presented as results of Tukey's multiple comparisons post hoc test following one-way ANOVA, performed using GraphPad Prism version 8.0.

Abbreviations: Mean Diff, mean difference between groups; 95% CI of diff, 95% confidence interval of the difference; Significant, statistical significance of the comparison; Summary, significance notation; Adjusted P Value, p-value corrected for multiple comparisons.

Statistical significance is indicated as follows: *p < 0.05, **p < 0.01 and ***p < 0.001.

Oxidative stress analysis in macrophages

Table S8. Tukey's Multiple Comparisons Test for Group Differences Obtained Using GraphPad Prism 8.0

Tukey's multiple comparisons test	Mean Diff,	95,00% CI of diff,	Significant?	Summary	Adjusted P Value
		**ROS levels**			
CONTROL vs. S180	25917	8557 to 43278	Yes	**	0,0020
CONTROL vs. VCR	12385	-4975 to 29746	No	ns	0,2446
CONTROL vs. 4-PSQ	17315	-45,68 to 34675	No	ns	0,0508
CONTROL vs. VCR + 4-PSQ	17292	-68,81 to 34652	No	ns	0,0512
S180 vs. VCR	-13532	-30893 to 3828	No	ns	0,1758
S180 vs. 4-PSQ	-8603	-25963 to 8758	No	ns	0,5846
S180 vs. VCR + 4-PSQ	-8626	-25986 to 8735	No	ns	0,5822
VCR vs. 4-PSQ	4930	-12431 to 22290	No	ns	0,9116
VCR vs. VCR + 4-PSQ	4907	-12454 to 22267	No	ns	0,9129
4-PSQ vs. VCR + 4-PSQ	-23,13	-17384 to 17337	No	ns	>0,9999
		**SOD activity**			
CONTROL vs. S180	1362	387,7 to 2336	Yes	**	0,0037
CONTROL vs. VCR	510,9	-463,2 to 1485	No	ns	0,5324
CONTROL vs. 4-PSQ	589,0	-385,1 to 1563	No	ns	0,3958
CONTROL vs. VCR + 4-PSQ	113,3	-860,8 to 1087	No	ns	0,9966
S180 vs. VCR	-850,9	-1825 to 123,1	No	ns	0,1054
S180 vs. 4-PSQ	-772,8	-1747 to 201,3	No	ns	0,1635
S180 vs. VCR + 4-PSQ	-1249	-2223 to -274,5	Yes	**	0,0081
VCR vs. 4-PSQ	78,12	-895,9 to 1052	No	ns	0,9992
VCR vs. VCR + 4-PSQ	-397,6	-1372 to 576,5	No	ns	0,7394
4-PSQ vs. VCR + 4-PSQ	-475,7	-1450 to 498,3	No	ns	0,5975
4-PSQ vs. VCR + 4-PSQ	-475,7	-1450 to 498,3	No	ns	0,5975
		**Sulfhydryl content**			
CONTROL vs. S180	1009	286,6 to 1731	Yes	**	0,0038
CONTROL vs. VCR	798,1	76,12 to 1520	Yes	*	0,0258
CONTROL vs. 4-PSQ	566,0	-155,9 to 1288	No	ns	0,1717
CONTROL vs. VCR + 4-PSQ	94,81	-627,1 to 816,8	No	ns	0,9945
S180 vs. VCR	-210,5	-932,4 to 511,5	No	ns	0,9037
S180 vs. 4-PSQ	-442,5	-1164 to 279,4	No	ns	0,3827
S180 vs. VCR + 4-PSQ	-913,7	-1636 to -191,8	Yes	**	0,0091
VCR vs. 4-PSQ	-232,0	-954,0 to 489,9	No	ns	0,8688
VCR vs. VCR + 4-PSQ	-703,3	-1425 to 18,68	No	ns	0,0585
4-PSQ vs. VCR + 4-PSQ	-471,2	-1193 to 250,7	No	ns	0,3233
4-PSQ vs. VCR + 4-PSQ	-471,2	-1193 to 250,7	No	ns	0,3233
		**CAT activity**			
CONTROL vs. S180	32,30	9,985 to 54,62	Yes	**	0,0027
CONTROL vs. VCR	8,766	-13,55 to 31,08	No	ns	0,7648
CONTROL vs. 4-PSQ	6,654	-15,66 to 28,97	No	ns	0,8964
CONTROL vs. VCR + 4-PSQ	4,157	-18,16 to 26,47	No	ns	0,9797
S180 vs. VCR	-23,53	-45,85 to -1,219	Yes	*	0,0356
S180 vs. 4-PSQ	-25,65	-47,96 to -3,332	Yes	*	0,0195
S180 vs. VCR + 4-PSQ	-28,14	-50,46 to -5,829	Yes	**	0,0093

VCR vs. 4-PSQ	-2,113	-24,43 to 20,20	No	ns	0,9985
VCR vs. VCR + 4-PSQ	-4,610	-26,92 to 17,71	No	ns	0,9705
4-PSQ vs. VCR + 4-PSQ	-2,497	-24,81 to 19,82	No	ns	0,9971
VCR vs. VCR + 4-PSQ	-4,610	-26,92 to 17,71	No	ns	0,9705
4-PSQ vs. VCR + 4-PSQ	-2,497	-24,81 to 19,82	No	ns	0,9971
**GST activity**					
CONTROL vs. S180	1424	-4479 to 7326	No	ns	0,9479
CONTROL vs. VCR	1126	-4777 to 7028	No	ns	0,9774
CONTROL vs. 4-PSQ	4432	-1471 to 10334	No	ns	0,2017
CONTROL vs. VCR + 4-PSQ	1418	-4484 to 7321	No	ns	0,9486
S180 vs. VCR	-298,1	-5863 to 5267	No	ns	0,9998
S180 vs. 4-PSQ	3008	-2557 to 8573	No	ns	0,5001
S180 vs. VCR + 4-PSQ	-5,747	-5571 to 5559	No	ns	>0,9999
VCR vs. 4-PSQ	3306	-2259 to 8871	No	ns	0,4093
VCR vs. VCR + 4-PSQ	292,4	-5273 to 5857	No	ns	0,9998
4-PSQ vs. VCR + 4-PSQ	-3014	-8579 to 2551	No	ns	0,4983
4-PSQ vs. VCR + 4-PSQ	-3014	-8579 to 2551	No	ns	0,4983
**Nitrite levels**					
CONTROL vs. S180	-0,08300	-0,1603 to -0,005716	Yes	*	0,0315
CONTROL vs. VCR	-0,02820	-0,1055 to 0,04908	No	ns	0,8086
CONTROL vs. 4-PSQ	0,02420	-0,05308 to 0,1015	No	ns	0,8791
CONTROL vs. VCR + 4-PSQ	0,03400	-0,04328 to 0,1113	No	ns	0,6846
S180 vs. VCR	0,05480	-0,02248 to 0,1321	No	ns	0,2497
S180 vs. 4-PSQ	0,1072	0,02992 to 0,1845	Yes	**	0,0040
S180 vs. VCR + 4-PSQ	0,1170	0,03972 to 0,1943	Yes	**	0,0017
VCR vs. 4-PSQ	0,05240	-0,02488 to 0,1297	No	ns	0,2887
VCR vs. VCR + 4-PSQ	0,06220	-0,01508 to 0,1395	No	ns	0,1539
4-PSQ vs. VCR + 4-PSQ	0,009800	-0,06748 to 0,08708	No	ns	0,9952
4-PSQ vs. VCR + 4-PSQ	0,009800	-0,06748 to 0,08708	No	ns	0,9952

Data are presented as results of Tukey's multiple comparisons post hoc test following one-way ANOVA, performed using GraphPad Prism version 8.0.

Abbreviations: Mean Diff, mean difference between groups; 95% CI of diff, 95% confidence interval of the difference; Significant, statistical significance of the comparison; Summary, significance notation; Adjusted P Value, p-value corrected for multiple comparisons.

Statistical significance is indicated as follows: *$p < 0.05$ and **$p < 0.01$.

Biochemical Assays

TBARS levels

Table S9. Tukey's Multiple Comparisons Test for Group Differences Obtained Using GraphPad Prism 8.0

Tukey's multiple comparisons test	Mean Diff,	95,00% CI of diff,	Significant?	Summary	Adjusted P Value
**Cerebral Cortex**					
CONTROL vs. S180	-96,65	-176,5 to -16,76	Yes	*	0,0122
CONTROL vs. VCR	-82,07	-162,0 to -2,183	Yes	*	0,0420
CONTROL vs. 4-PSQ	16,02	-63,87 to 95,90	No	ns	0,9755
CONTROL vs. VCR + PSQ	-12,09	-91,98 to 67,80	No	ns	0,9914
S180 vs. VCR	14,58	-65,31 to 94,46	No	ns	0,9827
S180 vs. 4-PSQ	112,7	32,77 to 192,6	Yes	**	0,0029
S180 vs. VCR + PSQ	84,56	4,670 to 164,4	Yes	*	0,0342
VCR vs. 4-PSQ	98,09	18,20 to 178,0	Yes	*	0,0108

VCR vs. VCR + PSQ	69,98	-9,905 to 149,9	No	ns	0,1065
4-PSQ vs. VCR + PSQ	-28,10	-108,0 to 51,79	No	ns	0,8376
**Spinal Cord**					
CONTROL vs. S180	-157,6	-259,2 to -56,01	Yes	**	0,0010
CONTROL vs. VCR	-128,7	-230,3 to -27,08	Yes	**	0,0082
CONTROL vs. 4-PSQ	-0,1753	-101,8 to 101,4	No	ns	>0,9999
CONTROL vs. VCR + 4-PSQ	-16,07	-117,7 to 85,52	No	ns	0,9898
S180 vs. VCR	28,93	-72,67 to 130,5	No	ns	0,9167
S180 vs. 4-PSQ	157,4	55,83 to 259,0	Yes	**	0,0010
S180 vs. VCR + 4-PSQ	141,5	39,94 to 243,1	Yes	**	0,0033
VCR vs. 4-PSQ	128,5	26,91 to 230,1	Yes	**	0,0083
VCR vs. VCR + 4-PSQ	112,6	11,01 to 214,2	Yes	*	0,0246
4-PSQ vs. VCR + 4-PSQ	-15,90	-117,5 to 85,70	No	ns	0,9902
CONTROL vs. S180	-157,6	-259,2 to -56,01	Yes	**	0,0010
**Hippocampus**					
CONTROL vs. S180	-10,28	-25,35 to 4,778	No	ns	0,2925
CONTROL vs. VCR	0,3446	-14,72 to 15,41	No	ns	>0,9999
CONTROL vs. 4-PSQ	8,230	-6,833 to 23,29	No	ns	0,5083
CONTROL vs. VCR + 4-PSQ	7,366	-7,696 to 22,43	No	ns	0,6111
S180 vs. VCR	10,63	-4,433 to 25,69	No	ns	0,2629
S180 vs. 4-PSQ	18,51	3,452 to 33,58	Yes	*	0,0107
S180 vs. VCR + 4-PSQ	17,65	2,589 to 32,71	Yes	*	0,0159
VCR vs. 4-PSQ	7,885	-7,178 to 22,95	No	ns	0,5491
VCR vs. VCR + 4-PSQ	7,021	-8,041 to 22,08	No	ns	0,6522
4-PSQ vs. VCR + 4-PSQ	-0,8634	-15,93 to 14,20	No	ns	0,9998
CONTROL vs. S180	-10,28	-25,35 to 4,778	No	ns	0,2925
**Cerebellum**					
CONTROL vs. S180	-155,1	-314,0 to 3,799	No	ns	0,0581
CONTROL vs. VCR	-95,77	-254,7 to 63,15	No	ns	0,4124
CONTROL vs. 4-PSQ	12,09	-146,8 to 171,0	No	ns	0,9994
CONTROL vs. VCR + 4-PSQ	4,782	-154,1 to 163,7	No	ns	>0,9999
S180 vs. VCR	59,35	-99,57 to 218,3	No	ns	0,8065
S180 vs. 4-PSQ	167,2	8,292 to 326,1	Yes	*	0,0357
S180 vs. VCR + 4-PSQ	159,9	0,9825 to 318,8	Yes	*	0,0481
VCR vs. 4-PSQ	107,9	-51,06 to 266,8	No	ns	0,2981
VCR vs. VCR + 4-PSQ	100,5	-58,37 to 259,5	No	ns	0,3647
4-PSQ vs. VCR + 4-PSQ	-7,310	-166,2 to 151,6	No	ns	>0,9999
CONTROL vs. S180	-155,1	-314,0 to 3,799	No	ns	0,0581
CONTROL vs. VCR	-95,77	-254,7 to 63,15	No	ns	0,4124

Data are presented as results of Tukey's multiple comparisons post hoc test following one-way ANOVA, performed using GraphPad Prism version 8.0.

Abbreviations: Mean Diff, mean difference between groups; 95% CI of diff, 95% confidence interval of the difference; Significant, statistical significance of the comparison; Summary, significance notation; Adjusted P Value, p-value corrected for multiple comparisons.

Statistical significance is indicated as follows: *p < 0.05 and **p < 0.01.

Na^+, K^+ - ATPase activity

Table S10. Tukey's Multiple Comparisons Test for Group Differences Obtained Using GraphPad Prism 8.0

Tukey's multiple comparisons test	Mean Diff,	95,00% CI of diff,	Significant?	Summary	Adjusted P Value
**Cerebral Cortex**					

CONTROL vs. S180	-8,692	-15,96 to -1,421	Yes	*	0,0135
CONTROL vs. VCR	1,381	-5,890 to 8,652	No	ns	0,9799
CONTROL vs. 4-PSQ	-8,510	-15,78 to -1,240	Yes	*	0,0161
CONTROL vs. VCR + PSQ	-2,123	-9,394 to 5,148	No	ns	0,9095
S180 vs. VCR	10,07	2,802 to 17,34	Yes	**	0,0035
S180 vs. 4-PSQ	0,1815	-7,089 to 7,452	No	ns	>0,9999
S180 vs. VCR + PSQ	6,569	-0,7018 to 13,84	No	ns	0,0906
VCR vs. 4-PSQ	-9,891	-17,16 to -2,621	Yes	**	0,0042
VCR vs. VCR + PSQ	-3,504	-10,77 to 3,767	No	ns	0,6239
4-PSQ vs. VCR + PSQ	6,387	-0,8833 to 13,66	No	ns	0,1049
		**Spinal Cord**			
CONTROL vs. S180	-0,4778	-5,151 to 4,195	No	ns	0,9981
CONTROL vs. VCR	2,953	-1,720 to 7,626	No	ns	0,3659
CONTROL vs. 4-PSQ	0,6397	-4,033 to 5,313	No	ns	0,9941
CONTROL vs. VCR + 4-PSQ	1,810	-2,863 to 6,483	No	ns	0,7853
S180 vs. VCR	3,431	-1,242 to 8,104	No	ns	0,2289
S180 vs. 4-PSQ	1,118	-3,556 to 5,791	No	ns	0,9540
S180 vs. VCR + 4-PSQ	2,288	-2,385 to 6,961	No	ns	0,6101
VCR vs. 4-PSQ	-2,313	-6,986 to 2,360	No	ns	0,6003
VCR vs. VCR + 4-PSQ	-1,143	-5,816 to 3,530	No	ns	0,9503
4-PSQ vs. VCR + 4-PSQ	1,170	-3,503 to 5,844	No	ns	0,9460
CONTROL vs. S180	-0,4778	-5,151 to 4,195	No	ns	0,9981
		**Hippocampus**			
CONTROL vs. S180	-2,259	-5,101 to 0,5833	No	ns	0,1675
CONTROL vs. VCR	1,748	-1,094 to 4,590	No	ns	0,3923
CONTROL vs. 4-PSQ	-0,2898	-3,132 to 2,552	No	ns	0,9981
CONTROL vs. VCR + 4-PSQ	0,8233	-2,019 to 3,665	No	ns	0,9119
S180 vs. VCR	4,007	1,165 to 6,849	Yes	**	0,0029
S180 vs. 4-PSQ	1,969	-0,8731 to 4,811	No	ns	0,2793
S180 vs. VCR + 4-PSQ	3,082	0,2400 to 5,924	Yes	*	0,0288
VCR vs. 4-PSQ	-2,038	-4,880 to 0,8043	No	ns	0,2490
VCR vs. VCR + 4-PSQ	-0,9249	-3,767 to 1,917	No	ns	0,8720
4-PSQ vs. VCR + 4-PSQ	1,113	-1,729 to 3,955	No	ns	0,7787
CONTROL vs. S180	-2,259	-5,101 to 0,5833	No	ns	0,1675
		**Cerebellum**			
CONTROL vs. S180	2,021	-4,865 to 8,907	No	ns	0,9080
CONTROL vs. VCR	3,487	-3,399 to 10,37	No	ns	0,5799
CONTROL vs. 4-PSQ	0,4670	-6,419 to 7,353	No	ns	0,9996
CONTROL vs. VCR + 4-PSQ	0,5780	-6,308 to 7,464	No	ns	0,9991
S180 vs. VCR	1,466	-5,421 to 8,352	No	ns	0,9696
S180 vs. 4-PSQ	-1,554	-8,440 to 5,332	No	ns	0,9625
S180 vs. VCR + 4-PSQ	-1,443	-8,329 to 5,443	No	ns	0,9712
VCR vs. 4-PSQ	-3,020	-9,906 to 3,866	No	ns	0,7008
VCR vs. VCR + 4-PSQ	-2,909	-9,795 to 3,977	No	ns	0,7283
4-PSQ vs. VCR + 4-PSQ	0,1110	-6,775 to 6,997	No	ns	>0,9999
CONTROL vs. S180	2,021	-4,865 to 8,907	No	ns	0,9080
CONTROL vs. VCR	3,487	-3,399 to 10,37	No	ns	0,5799

Data are presented as results of Tukey’s multiple comparisons post hoc test following one-way ANOVA, performed using GraphPad Prism version 8.0.

Abbreviations: Mean Diff, mean difference between groups; 95% CI of diff, 95% confidence interval of the difference; Significant, statistical significance of the comparison; Summary, significance notation; Adjusted P Value, p-value corrected for multiple comparisons.

Statistical significance is indicated as follows: *$p < 0.05$ and **$p < 0.01$.

Mg^{2+} - ATPase activity

Table S11. Tukey’s Multiple Comparisons Test for Group Differences Obtained Using GraphPad Prism 8.0

Tukey's multiple comparisons test	Mean Diff,	95,00% CI of diff,	Significant?	Summary	Adjusted P Value
**Cerebral Cortex**					
CONTROL vs. S180	5,521	-4,799 to 15,84	No	ns	0,5308
CONTROL vs. VCR	14,98	4,267 to 25,69	Yes	**	0,0031
CONTROL vs. 4-PSQ	4,930	-5,780 to 15,64	No	ns	0,6648
CONTROL vs. VCR + PSQ	6,435	-4,274 to 17,15	No	ns	0,4173
S180 vs. VCR	9,456	-0,8646 to 19,78	No	ns	0,0842
S180 vs. 4-PSQ	-0,5915	-10,91 to 9,729	No	ns	0,9998
S180 vs. VCR + PSQ	0,9141	-9,406 to 11,23	No	ns	0,9989
VCR vs. 4-PSQ	-10,05	-20,76 to 0,6627	No	ns	0,0737
VCR vs. VCR + PSQ	-8,542	-19,25 to 2,168	No	ns	0,1659
4-PSQ vs. VCR + PSQ	1,506	-9,204 to 12,22	No	ns	0,9936
**Spinal Cord**					
CONTROL vs. S180	23,20	-3,403 to 49,80	No	ns	0,1089
CONTROL vs. VCR	22,20	-4,403 to 48,80	No	ns	0,1348
CONTROL vs. 4-PSQ	3,106	-23,49 to 29,70	No	ns	0,9968
CONTROL vs. VCR + 4-PSQ	-12,69	-39,29 to 13,91	No	ns	0,6328
S180 vs. VCR	-1,000	-27,60 to 25,60	No	ns	>0,9999
S180 vs. 4-PSQ	-20,09	-46,69 to 6,509	No	ns	0,2058
S180 vs. VCR + 4-PSQ	-35,88	-62,48 to -9,285	Yes	**	0,0045
VCR vs. 4-PSQ	-19,09	-45,69 to 7,509	No	ns	0,2482
VCR vs. VCR + 4-PSQ	-34,88	-61,48 to -8,285	Yes	**	0,0059
4-PSQ vs. VCR + 4-PSQ	-15,79	-42,39 to 10,81	No	ns	0,4269
CONTROL vs. S180	23,20	-3,403 to 49,80	No	ns	0,1089
**Hippocampus**					
CONTROL vs. S180	-3,776	-8,621 to 1,070	No	ns	0,1819
CONTROL vs. VCR	6,228	1,383 to 11,07	Yes	**	0,0072
CONTROL vs. 4-PSQ	-0,7062	-5,552 to 4,139	No	ns	0,9926
CONTROL vs. VCR + 4-PSQ	0,01734	-4,828 to 4,863	No	ns	>0,9999
S180 vs. VCR	10,00	5,159 to 14,85	Yes	****	<0,0001
S180 vs. 4-PSQ	3,070	-1,776 to 7,915	No	ns	0,3635
S180 vs. VCR + 4-PSQ	3,793	-1,052 to 8,639	No	ns	0,1785
VCR vs. 4-PSQ	-6,935	-11,78 to -2,089	Yes	**	0,0025
VCR vs. VCR + 4-PSQ	-6,211	-11,06 to -1,366	Yes	**	0,0074
4-PSQ vs. VCR + 4-PSQ	0,7235	-4,122 to 5,569	No	ns	0,9918
CONTROL vs. S180	-3,776	-8,621 to 1,070	No	ns	0,1819
**Cerebellum**					
CONTROL vs. S180	-12,38	-21,40 to -3,366	Yes	**	0,0038
CONTROL vs. VCR	1,102	-7,916 to 10,12	No	ns	0,9962
CONTROL vs. 4-PSQ	0,4517	-8,566 to 9,470	No	ns	0,9999
CONTROL vs. VCR + 4-PSQ	-6,469	-15,49 to 2,549	No	ns	0,2486
S180 vs. VCR	13,49	4,469 to 22,50	Yes	**	0,0015
S180 vs. 4-PSQ	12,84	3,818 to 21,85	Yes	**	0,0026
S180 vs. VCR + 4-PSQ	5,916	-3,102 to 14,93	No	ns	0,3300
VCR vs. 4-PSQ	-0,6505	-9,668 to 8,367	No	ns	0,9995
VCR vs. VCR + 4-PSQ	-7,571	-16,59 to 1,447	No	ns	0,1311
4-PSQ vs. VCR + 4-PSQ	-6,920	-15,94 to 2,098	No	ns	0,1934
CONTROL vs. S180	-12,38	-21,40 to -3,366	Yes	**	0,0038
CONTROL vs. VCR	1,102	-7,916 to 10,12	No	ns	0,9962

Data are presented as results of Tukey's multiple comparisons post hoc test following one-way ANOVA, performed using GraphPad Prism version 8.0.

Abbreviations: Mean Diff, mean difference between groups; 95% CI of diff, 95% confidence interval of the difference; Significant, statistical significance of the comparison; Summary, significance notation; Adjusted P Value, p-value corrected for multiple comparisons.

Statistical significance is indicated as follows: **$p < 0.01$ and ****$p < 0.0001$.

General Procedure for the Synthesis of 7-chloro-4-(phenylselanyl)quinoline

To a round-bottomed flask containing a solution of diphenyl diselenide (0.25 mmol) in PEG-400 (1.0 mL) under N_2 atmosphere, was added $NaBH_4$ (1.0 mmol). The resulting solution was stirred for 1 hour at 60 °C, when its color changes from yellow to colorless. After this, 4,7-dichloroquinoline (0.5 mmol) was added and the mixture was stirred at 60 °C for additional 1 hour. Then, the reaction mixture was received in water (10 mL), extracted with ethyl acetate (3 x 5 mL), dried over $MgSO_4$, and concentrated under vacuum. The residue was purified by column chromatography on silica gel using a mixture of ethyl acetate/hexanes (20:80) as the eluent.

Spectral and analytical data for: 7-chloro-4-(phenylselanyl)quinoline: Yield: 0.156 g (98%); pale yellow solid; mp 81-83 °C. ^{1}H NMR (400 MHz, $CDCl_3$); δ (ppm): 8.51 (d, J = 4.7 Hz, 1H), 8.07 (d, J = 2.0 Hz, 1H), 7.99 (d, J = 8.9 Hz, 1H), 7.66-7.63 (m, 2H), 7.53-7.42 (m, 4H), 6.97 (d, J = 4.7 Hz, 1H). ^{13}C NMR (100 MHz, $CDCl_3$) δ = 150.27, 147.97, 145.89, 136.16, 135.44, 129.95, 129.34, 128.75, 127.39, 126.48, 126.07, 125.82, 121.70. MS (relative intensity) *m/z*: 321 (42), 320 (21), 319 (100), 317 (49), 284 (33), 282 (18), 241 (25), 239 (77), 204 (70), 162 (18), 142 (15), 135 (30), 127 (29), 99 (44), 77 (29), 75 (11), 51 (25). HRMS calcd. for $C_{15}H_{11}ClNSe$: $[M+H]^+$ 319.97452. Found: 319.97546.

Chromatogram BD10 C:\Users\Usuario\Desktop\QSePh.qgd

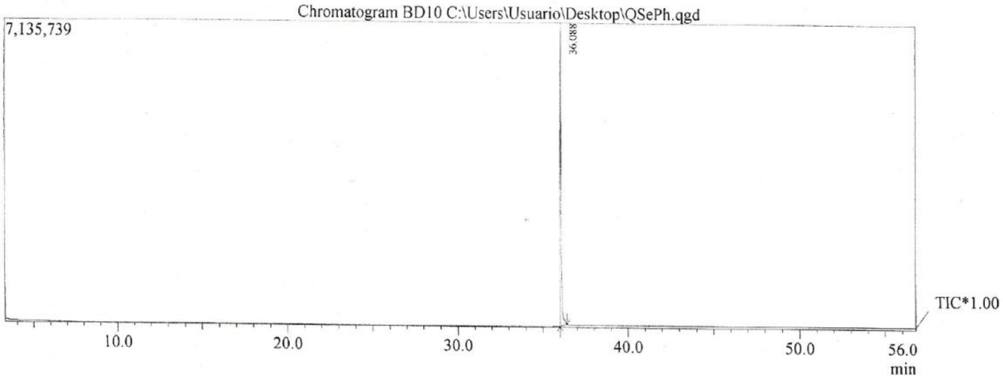

Peak Report TIC

Peak#	R.Time	I.Time	F.Time	Area	Area%	Height	Height%	A/H	Mark	Name
1	36.088	36.000	36.417	31558122	100.00	7099734	100.00	4.44	MI	
				31558122	100.00	7099734	100.00			

Spectrum

Line#:1 R.Time:36.1(Scan#:3934)
MassPeaks:44
RawMode:Averaged 36.1-36.1(3931-3935) BasePeak:319(537230)
BG Mode:Averaged 35.3-35.4(3839-3854) Group 1 - Event 1

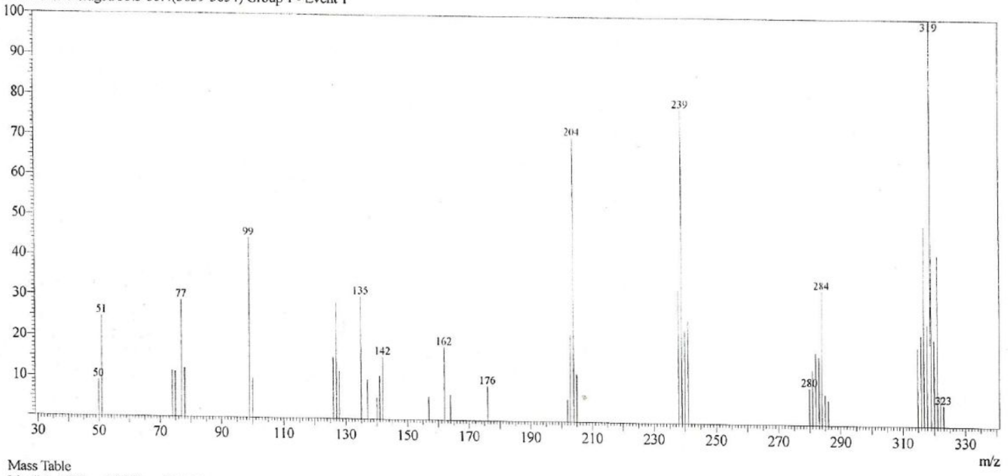

Mass Table
Line#:1 R.Time:36.1(Scan#:3934)
MassPeaks:44
RawMode:Averaged 36.1-36.1(3931-3935) BasePeak:319(537230)
BG Mode:Averaged 35.3-35.4(3839-3854) Group 1 - Event 1

#	m/z	Abs. Int.	Rel. Int.	#	m/z	Abs. Int.	Rel. Int.	#	m/z	Abs. Int.	Rel. Int.
1	49.95	48426	9.01	16	142.00	82399	15.34	31	281.95	95317	17.74
2	50.95	132734	24.71	17	156.95	30474	5.67	32	282.95	90351	16.82
3	73.95	61260	11.40	18	162.00	96238	17.91	33	284.00	176143	32.79
4	74.95	60576	11.28	19	164.00	33523	6.24	34	285.00	40508	7.54
5	77.00	154640	28.78	20	176.00	45906	8.54	35	286.00	32666	6.08
6	78.00	64916	12.08	21	202.00	29908	5.57	36	314.95	102864	19.15
7	99.00	238523	44.40	22	203.00	115437	21.49	37	315.95	120015	22.34
8	100.00	52541	9.78	23	204.05	377342	70.24	38	316.95	264033	49.15
9	126.05	81842	15.23	24	205.05	64363	11.98	39	317.95	133968	24.94
10	127.05	154740	28.80	25	238.00	175874	32.74	40	319.00	537230	100.00
11	128.00	63756	11.87	26	239.00	415396	77.32	41	320.00	113887	21.20
12	135.00	161646	30.09	27	240.00	122724	22.84	42	321.00	224889	41.86
13	137.00	52471	9.77	28	241.00	135141	25.16	43	321.95	40775	7.59
14	140.05	28564	5.32	29	279.95	48817	9.09	44	322.95	27830	5.18
15	141.00	58010	10.80	30	280.95	73324	13.65				

Figure S1. Chromatogram and Mass Spectrum for **7-chloro-4-(phenylselanyl)quinoline**.

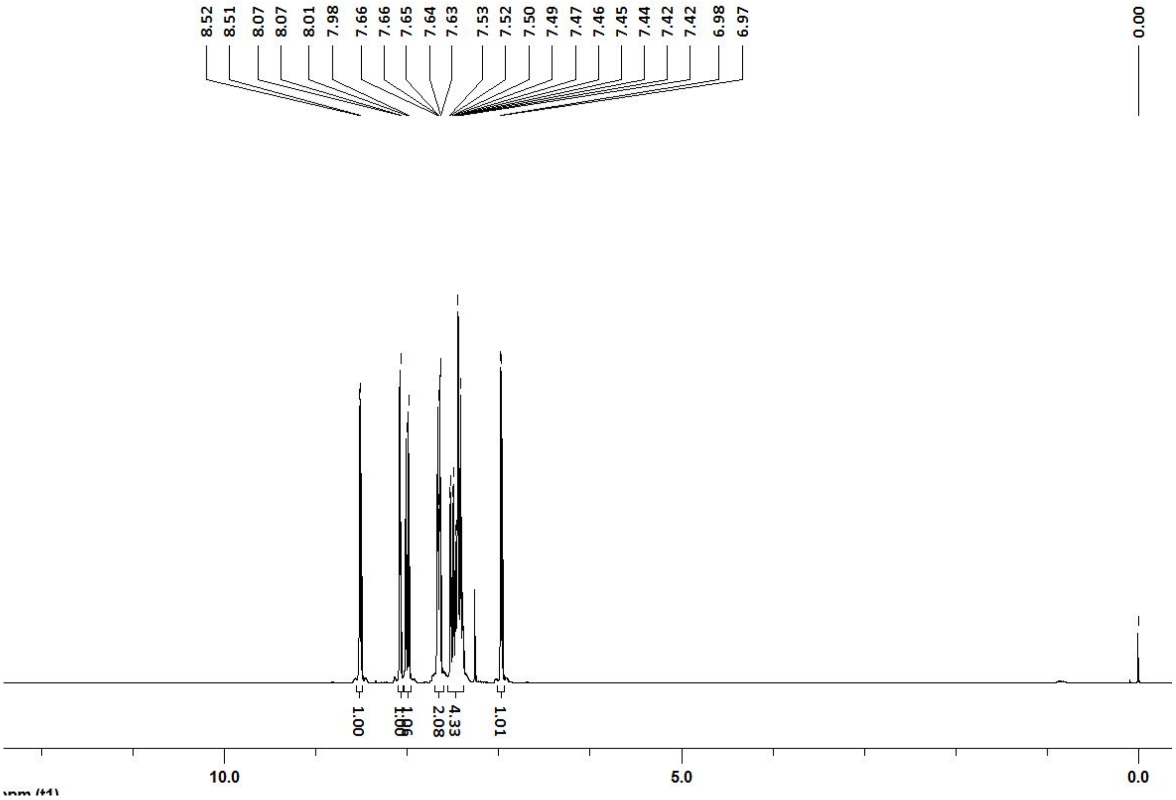

Figure S2. ^{1}H NMR (400 MHz) spectrum for **7-chloro-4-(phenylselanyl)quinoline** in $CDCl_3$.

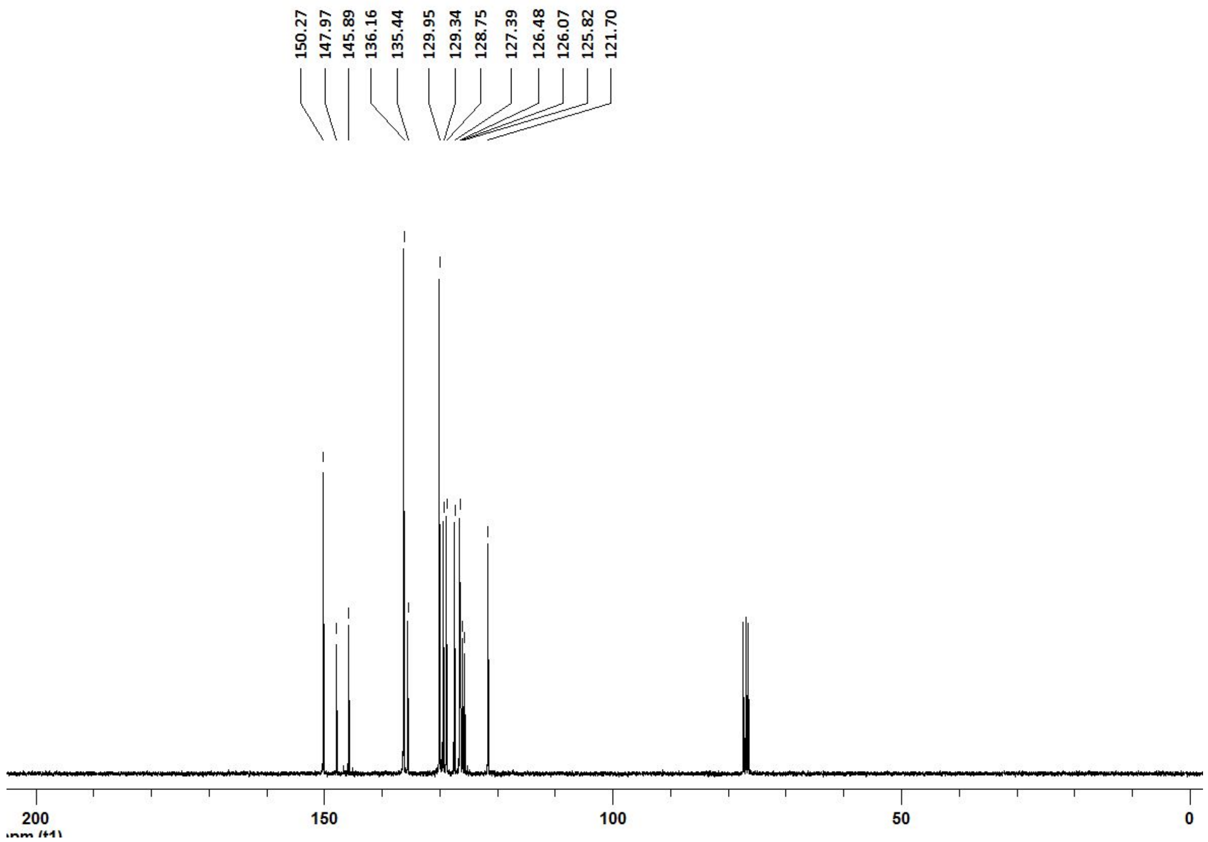

Figure S3. ^{13}C NMR (100 MHz) spectrum for **7-chloro-4-(phenylselanyl)quinoline** in $CDCl_3$.

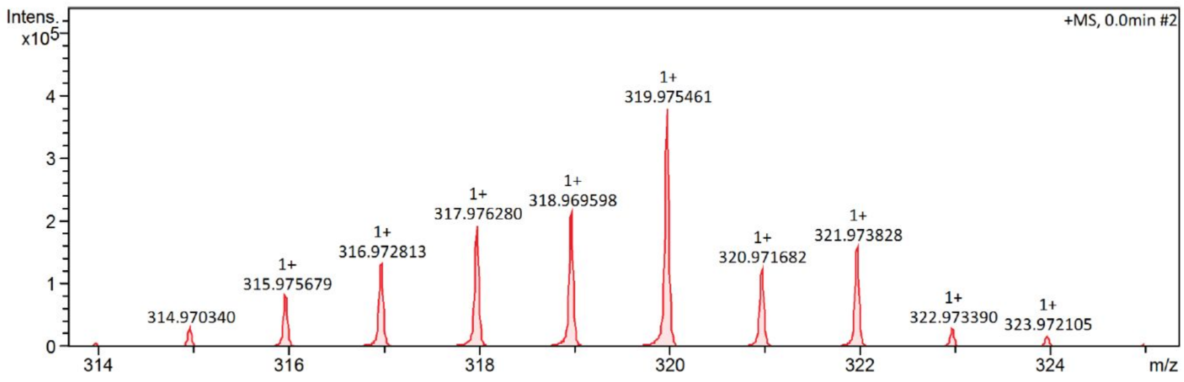

Figure S4. HRMS spectrum for **7-chloro-4-(phenylselanyl)quinoline**.
